# Acute axon damage and demyelination are mitigated by 4-aminopyridine (4-AP) therapy after experimental traumatic brain injury

**DOI:** 10.1186/s40478-022-01366-z

**Published:** 2022-05-02

**Authors:** Kryslaine L. Radomski, Xiaomei Zi, Fritz W. Lischka, Mark D. Noble, Zygmunt Galdzicki, Regina C. Armstrong

**Affiliations:** 1 Department of Anatomy, Physiology and Genetics, Uniformed Services University of the Health Sciences, 4301 Jones Bridge Road, Bethesda, MD 20814 USA; 2 Center for Neuroscience and Regenerative Medicine, Uniformed Services University of the Health Sciences, 4301 Jones Bridge Road, Bethesda, MD 20814 USA; 3 Biomedical Instrumentation Center, Uniformed Services University of the Health Sciences, 4301 Jones Bridge Road, Bethesda, MD 20814 USA; 4 Department of Biomedical Genetics, School of Medicine and Dentistry, University of Rochester, 601 Elmwood Ave, Box 633, Rochester, NY 14642 USA

**Keywords:** Traumatic brain injury, 4-aminopyridine, Demyelination, White matter, Kv1.2, Electrophysiology

## Abstract

Damage to long axons in white matter tracts is a major pathology in closed head traumatic brain injury (TBI). Acute TBI treatments are needed that protect against axon damage and promote recovery of axon function to prevent long term symptoms and neurodegeneration. Our prior characterization of axon damage and demyelination after TBI led us to examine repurposing of 4-aminopyridine (4-AP), an FDA-approved inhibitor of voltage-gated potassium (Kv) channels. 4-AP is currently indicated to provide symptomatic relief for patients with chronic stage multiple sclerosis, which involves axon damage and demyelination. We tested clinically relevant dosage of 4-AP as an acute treatment for experimental TBI and found multiple benefits in corpus callosum axons. This randomized, controlled pre-clinical study focused on the first week after TBI, when axons are particularly vulnerable. 4-AP treatment initiated one day post-injury dramatically reduced axon damage detected by intra-axonal fluorescence accumulations in Thy1-YFP mice of both sexes. Detailed electron microscopy in C57BL/6 mice showed that 4-AP reduced pathological features of mitochondrial swelling, cytoskeletal disruption, and demyelination at 7 days post-injury. Furthermore, 4-AP improved the molecular organization of axon nodal regions by restoring disrupted paranode domains and reducing Kv1.2 channel dispersion. 4-AP treatment did not resolve deficits in action potential conduction across the corpus callosum, based on ex vivo electrophysiological recordings at 7 days post-TBI. Thus, this first study of 4-AP effects on axon damage in the acute period demonstrates a significant decrease in multiple pathological hallmarks of axon damage after experimental TBI.

## Introduction

Traumatic brain injury (TBI) results in long term disability in moderate to severe cases and can cause persistent debilitating symptoms even in patients with a “mild” diagnosis [41, 46, 53, 85]. Traumatic injury to long axons within white matter tracts is a key pathology in all forms of closed head TBI. Importantly, white matter injury represents a treatable target for TBI and neurodegenerative diseases, including multiple sclerosis (MS) and Alzheimer’s disease [2, 31, 64, 65].

The lack of treatments for TBI, particularly treatments to protect axons that do not effectively regenerate, is a critical gap in research and clinical care. At an early stage of damage, axons have the potential to recover but once mechanical or molecular processes fragment the axon, then irreversible axon degeneration proceeds [35, 48, 51, 52, 57, 79, 80]. Use of transgenic mice with fluorescent labeling of axons has shown that axonal swellings can appear within minutes of an insult and recover within hours, or persist for weeks or more, and can degenerate to form terminal end bulbs at sites of disconnection [35, 57, 59, 78]. Axons can also continue to initiate distal, or Wallerian, degeneration in the days to weeks after an initial mechanical injury [52]. Additionally, axons that remain viable can undergo demyelination, i.e. loss of their myelin sheaths, which impairs rapid signal conduction and desynchronizes neural circuits. Axon damage and demyelination contribute to slow information processing and neural circuit deficits that often underlie symptoms of mild-moderate TBI [16, 23, 27]. Maintaining axon and myelin function can alleviate symptoms and prevent irreversible white matter degeneration after TBI. No available treatments effectively protect against axon degeneration or promote remyelination, which protects axons and enhances recovery [44, 58].

Treatment strategies for TBI, which have failed to date, could gain from analysis of potential therapeutic targets focused on axon or myelin damage in the context of white matter pathology. Our studies of white matter injury revealed axon and myelin damage that included disruption of nodal regions and slowed axon conduction velocity early after TBI [50, 54]. These findings of impaired axon conduction and demyelination resemble aspects for which 4-aminopyridine (4-AP) was developed to treat patients with MS. 4-AP is prescribed for patients in the chronic stage of MS and responders have positive effects on walking ability, finger dexterity, and cognitive function [87]. Recent pre-clinical testing on acute peripheral nerve injuries, which also involve axon and myelin damage, revealed the surprising result that 4-AP treatment enhances structural recovery of axons and myelin [74]. Thus, 4-AP may be beneficial for acute traumatic injury and warrants evaluation of repurposing for TBI.

4-AP is a small molecule inhibitor of voltage-gated potassium (Kv) channels that readily crosses the blood–brain barrier yet has an uncertain mode of action in patients [20, 34]. In MS and other chronic neurological diseases, 4-AP is thought to enhance action potential conduction and modulate overall neural circuit activity through inhibiting aberrant Kv currents of axons and/or amplification of synaptic transmission [17, 69, 87]. Therapeutically, low dose (10 mg, twice daily) extended release 4-AP (dalfampridine) is effective at blood levels below 100 ng/ml (~ 1 µM) without unacceptable adverse effects, particularly seizure induction [34].

We conducted randomized, controlled pre-clinical studies to evaluate repurposing of 4-AP as an acute treatment for TBI. Low therapeutically relevant 4-AP dosing was initiated one day after TBI to evaluate axon integrity and electrophysiological function during the first week, when axons are particularly vulnerable. A single impact closed head TBI in adult mice produced traumatic axonal injury in the corpus callosum (CC). This injury models human TBI pathology and neuroimaging features from acute injury through chronic neurodegeneration [7, 50, 54, 72]. The current study design demonstrates reproducible CC pathology and functional deficits to rigorously evaluate acute effects of 4-AP treatment. We identify multiple positive effects of 4-AP treatment on mitigating axon pathology after TBI.

## Materials and methods

### Study design

**Fig. 1:**
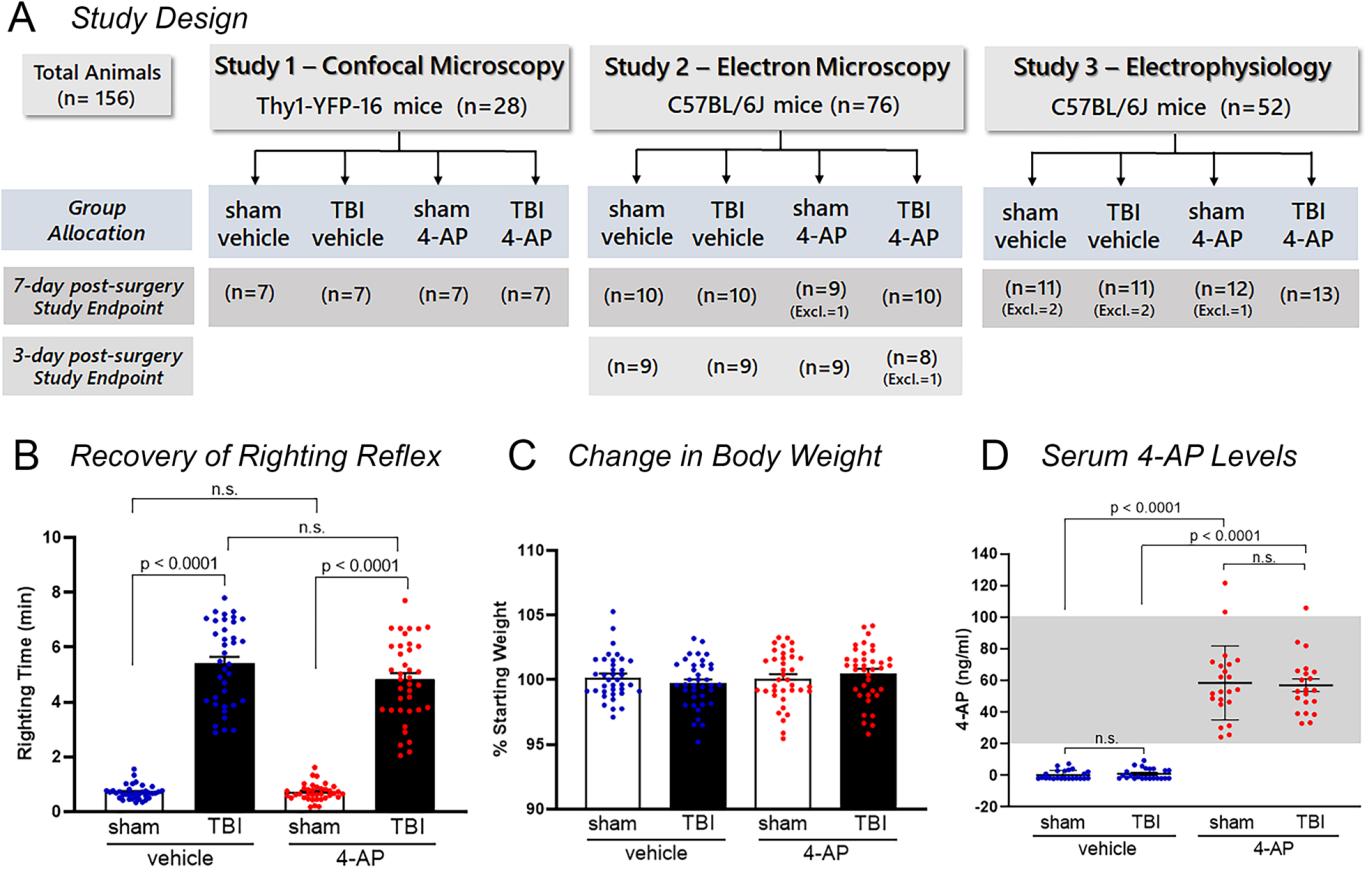
Experimental study design and key assessments of group allocation. **A** Graphical representation of experimental timeline and treatment groups. Three sets of experiments were conducted according to pre-determined study designs. For each study, the strain and number of mice used is shown along with the study endpoint and method of analysis of corpus callosum axons. Excl. = number of mice excluded based on pre-determined criteria (see Materials and Methods for definition). **B** TBI increased the time to right from supine to prone position immediately after surgery (righting reflex) as compared with respective sham groups. This confirms that mice randomized for allocation to the TBI vehicle and TBI 4-AP groups exhibited similar righting times prior to initiating 4-AP treatment. **C** 4-AP treatment did not cause a significant change in body weight over the period of the experiment. **D** 4-AP serum drug level concentrations are within the clinical therapeutic target range (20–100 ng/ml) in cardiac blood collected approximately 30–60 min after last 4-AP injection. Graphs show the mean ± SEM with each mouse as an individual data point. One-way ANOVA with Tukey’s multiple comparisons test: **B** F_3,145_ = 211.3, *p* < 0.0001, **C** F_3,145_ = 1.057, *p* = 0.3692. Two-way ANOVA with Holm-Sidak post hoc test: **D** Interaction: F_1,185_ = 0.1281, *p* = 0.7213; Injury F_1,185_ = 0.0183, *p* = 0.8927; Drug; F_1,185_ = 332.8, *p* < 0.0001

Experiments in this study (Fig. 1) were performed using a blinded, randomized, controlled trial design [38]. Animal procedures were designed and implemented according to the ARRIVE (Animal Research: Reporting In Vivo Experiments) guidelines [43]. Pre-determined study designs with details for data collection, standardized method protocols, outcome measures, sample size estimation (> 80% power), statistical analyses, and rules for data exclusion were pre-registered on Open Science Framework (https://osf.io/registries) prior to executing experiments. Each investigation followed a pre-determined study design to evaluate 4-AP treatment using three complementary approaches: (1) screen for axon damage and analyze the molecular organization at nodes of Ranvier by confocal microscopy using Thy1-YFP-16 reporter mice; (2) conduct in-depth axon-myelin pathology by electron microscopy (EM) using C57BL/6 J mice; and (3) directly examine axon function by ex vivo electrophysiological recordings using coronal brain slices from C57BL/6 J mice.

### Randomization and blinding procedures

All animals were subcutaneously implanted with a micro-transponder (IMI-500, Bio Medic Data Systems, Seaford, DE) two days before surgery and the designated alpha-numeric codes were used for randomization and animal group allocation prior to surgical procedures. Computer-generated randomization was achieved by first assigning mice to TBI or sham surgical groups, then to vehicle or 4-AP treatment groups using the RANDBETWEEN function in Microsoft Excel (RRID:SCR_016137). Both the investigators administering the drug/vehicle and those performing data analyses were blinded as to injury and treatment conditions. All data were collected and statistical analyses were completed prior to unblinding. The study designs, including the number of animals randomized to sham-vehicle, TBI-vehicle, sham-4-AP, or TBI-4-AP groups with endpoints at either 3- or 7-days post-injury are shown in Fig. 1A.

### Mice

At the start of experiments, mice were 8–10 weeks old, which approximately corresponds to young adulthood in humans [26], a patient population at high risk of TBI [73]. Transgenic mice expressing YFP under the control of the Thy-1 promoter (Thy1-YFP-16) on a C57BL/6 background were acquired from Jackson Laboratories (Bar Harbor, ME). The original Thy1-YFP-16 mice (IMSR Cat# JAX:003,709, RRID:IMSR_JAX:003,709; B6.Cg-Tg(Thy1-YFP)16Jrs/J) were bred in-house to generate the experimental male (n = 3/group) and female (n = 4/group) mice for Study 1 – Confocal Analysis. As no sex-based differences were detected in this initial study, the experiments of Studies 2 and 3 used only male mice. C57BL/6J (IMSR Cat# JAX:000,664, RRID:IMSR_JAX:000,664) mice were purchased from Jackson Laboratories. Mice were socially housed in 35 cm × 16.5 cm × 18 cm enrichment cages (2–5 mice per cage), exposed to 12:12 h light–dark cycle, at controlled room temperature, and with free access to food and water. All experimental procedures were conducted during the daytime light cycle (0600–1800) after the mice had acclimated for at least 3 days in their home cage.

### TBI model

A single impact closed head TBI model was used to produce traumatic axonal injury in the CC that models traumatic axonal injury in human TBI pathophysiology [7, 50, 54, 55, 72, 84]. An Impact One Stereotaxic Impactor (Leica Biosystems, Buffalo Grove, IL) attached to a steel impactor with a 3-mm flat tip with rounded edges (Cat# 2520-3S, Neuroscience Tools, Aurora, IL) was zeroed on the surface of the skull centered at bregma (0 ML, 0 AP, 0 DV). In the coronal plane, bregma is aligned with the mid-line crossing of the anterior commissure, which served to localize the injury site in coronal brain sections. For mice in the TBI groups, the impactor was set to 4 m/s with a dwell time of 0.1 s and a depth of 1.5 mm from the surface of the skull (0 ML, 0 AP,  − 1.5 DV). Isoflurane administration was terminated immediately prior to impact. Our prior studies have shown that these parameters induce mild-moderate closed skull TBI with CC pathology that progresses to late stage CC atrophy without gross tissue damage or microhemorrhages, detected by neuropathology or magnetic resonance imaging [7, 50, 54, 84]. Sham mice followed identical procedures but did not receive the impact. After scalp closure, mice were placed in a warm cage in the supine position to record the righting reflex as a measure of the time it takes each mouse to regain consciousness. A delay in the motor ability for a mouse to right itself from a supine position (righting reflex) immediately after the surgical procedure indicates a transient alteration of consciousness that is increased after TBI [19, 56].

### 4-Aminopyridine preparation and dosing

4-aminopyridine (4-AP, fampridine; Cat# 275,775; ≥ 99% purity; Sigma-Aldrich, St. Louis, MO) was dissolved in sterile saline (0.9% sodium chloride; Hanna Pharmaceutical Supply Co., Inc.; Cat# 0,409,488,810) to a final concentration of 0.1 µg/µl, and intraperitoneally (i.p.) injected at a dose of 0.5 mg/kg body weight. 4-AP dosing was calculated to approximate a relevant human clinical dose equivalent (clinical serum dose range of 20–100 ng/ml) as reported in prior rodent studies that have demonstrated 4-AP positive effects after peripheral nerve and pyramidal tract injuries [66, 74]. Mice were injected i.p. twice a day (11–13 h apart) with either 4-AP solution or equivalent volumes of saline. Mice were weighed at baseline (surgery day) and on each day to determine the appropriate dose. Injections were initiated at 24 h after sham or TBI procedures to align with a clinically reasonable interval for the time to first dose. The study endpoint was either 3- or 7-days post-sham or TBI procedures. For ex vivo electrophysiological studies, mice did not receive a final dose on day 7 post-injury in order to examine the endogenous electrophysiology without potential variation due to the in vivo 4-AP levels at the time of brain collection. Mice did not exhibit overt changes in coat, posture, or activity with 4-AP treatment. The percentage change in body weight at the study endpoint was calculated relative to each animal's baseline as a measure of overall health relative to 4-AP treatment or TBI.

### 4-AP quantification in serum

For analysis of 4-AP serum levels, mice received the last 4-AP or vehicle injection approximately 30–60 min before cardiac blood was collected under deep anesthesia just prior to transcardial perfusion. Blood was undisturbed at room temperature for a 1 h clotting time, and then serum was separated by centrifugation at 3,000 × g for 10 min and stored at  − 80 °C. 4-AP serum concentration was analyzed by liquid chromatography coupled with tandem mass spectrometry (LC–MS/MS) as previously detailed [39]. All data points are included in the analyses except for: (1) one cohort (n = 3/group) from the 3-day C57BL/6J mice (Study 2), which had blood collected at longer times after injection for analysis of drug washout; (2) one mouse from the 7-day C57BL/6J cohort (Study 2) for which we were unable to collect enough blood; and (3) all mice used for electrophysiological recordings since the experiment was designed with a one day washout period in order to record the endogenous electrophysiological function.

### Confocal microscopy

Thy1-YFP-16 reporter male and female mice were perfused with 4% paraformaldehyde (PFA). Collected brains were post-fixed in the same fixative overnight, cryoprotected and then cut as 14 µm coronal cryostat sections. The region-of-interest (ROI) was located in equivalent areas of the medial CC situated between the cingulum and the longitudinal fissure in coronal brain slices under the impact site. High resolution images were acquired on a Zeiss LSM 700 confocal microscope (RRID:SCR_017377) using either a 40x/1.4 or a 63x/1.4 oil Plan-Apochromat objective, for analyses of YFP+ accumulations and nodal domains respectively.

### Confocal analysis of axonal swellings in Thy1-YFP-16 mice

For each mouse, three separate confocal image stacks were analyzed and averaged to obtain a single value per mouse. The confocal image stacks were acquired with a volume of (mean ± SD) 219.8 ± 5.6 µm × 98.7 ± 13.0 µm × 4.90 µm (14 optical slices per stack) centered over the medial CC in either cerebral hemisphere. After conversion of image z-stacks to maximum intensity projections, images were processed using ImageJ software (NIH, RRID:SCR_003070) for conversion to grayscale and spatial calibration. In order to generate a binary mask for automated particle analysis, an adequate threshold was selected for each image based on an optimal separation of gray levels in swollen axons exhibiting strong YFP+ accumulations against background that included intact YFP-expressing axons. The ImageJ particle analysis plug-in was then used for quantification over the entire image (excluding edges). This process enabled automated quantification of the percent area containing YFP accumulations within the defined imaged volume. Results were analyzed for sex as a biological variable and as no significant differences were found, the data from males and females were combined.

### Confocal analysis of nodal and paranodal domains

Immunohistochemistry, confocal imaging, and quantification of paranodal complexes were described in detail previously [50]. Briefly, detection of Nav1.6 (Alomone Labs, Jerusalem, Israel; Cat# ASC-009, RRID:AB_2040202) at the nodes of Ranvier and Caspr (UC Davis/NIH NeuroMab Facility Cat# K65/35, RRID:AB_2877274) at adjacent paranodes. Nav1.6 and Caspr localization was detected on confocal images acquired with a volume of 67.74 µm × 67.74 µm × 4.98 µm using a 0.45 µm z-interval separation between image planes. For each mouse, one image stack per hemisphere was collected and analyzed. Paranode domain organization was assessed by manual length measurements of 100 randomly selected pairs of Caspr domains for each cerebral hemisphere (total of 200 Caspr domains per mouse) in the 2D plane using maximum intensity z-projections (MIP). The average length difference within paranode pairs (paranodal asymmetry), the nodal gap length (distance between paired Caspr immunolabeled domains), and the total number of heminodes (unpaired Caspr domain adjacent to Nav1.6 immunolabeled node region) were tabulated. Because YFP fluorescence in TBI-induced axonal swellings can readily differentiate injured from non-injured tissue sections from Thy1-YFP-16 mice, the initial analysis did not include the YFP channel in order to avoid potential bias. After quantification was completed, confocal z-stacks were re-assessed using the 3-dimensional viewing software arivis Vision 4D (RRID:SCR_018000) to confirm that the identified unpaired Caspr domains (heminodes) met the pre-determined criteria of expression within YFP-expressing axons as detailed in Marion et al. [50]. Only Caspr domains localized along YFP axons were included in the analysis.

### Confocal localization of juxtaparanodal Kv1.2 channel domain

Cryostat sections from Thy1-YFP-16 mice were subjected to antigen retrieval with Tris–EDTA buffer (pH 9.0) in a 90 °C water bath for 15 min. Co-labeling with primary antibodies for the juxtaparanode marker Kv1.2 (UC Davis/NIH NeuroMab Facility Cat# K14/16, RRID:AB_2877295) and the paranode marker Caspr (Abcam, Cambridge, UK; Cat# ab34151, RRID:AB_869934) was conducted overnight at 4 °C. Sections were then exposed overnight to secondary antibodies donkey anti-mouse Alexa Fluor 594 (Cat# 715–586-151, RRID:AB_2340858) and donkey anti-rabbit Alexa Fluor 647 (Cat# 711–606-152, RRID:AB_2340625) both from Jackson ImmunoResearch (West Grove, PA). Kv1.2 and Caspr localization was analyzed on confocal image stacks of 11 µm in depth and measuring (mean ± SD) 131.0 ± 66.2 µm × 13.6 ± 15.9 µm in an area over the medial CC. Given the sensitivity of YFP expression to axon damage, Kv1.2 and Caspr domains were first analyzed without the YFP channel visible in order to maintain the investigator blinded to surgical group allocation. ImageJ Cell Counter plugin was used to classify all identifiable Kv1.2 immunolabeled juxtaparanodal domains within the MIP image as either “typical” or “atypical”. Typical Kv1.2 expression did not overlap with Caspr immunolabeled paranodal domains and appeared symmetrical in length. Atypical Kv1.2 expression exhibited > 30% overlap with the Caspr domain and/or appeared asymmetrical, often with Kv1.2 dispersion away from the nodal region into the internode. After completion, each classified Kv1.2 nodal complex was reanalyzed by sequentially examining individual optical planes with the YFP signal on using the Zeiss Zen Black software (ZEISS, RRID:SCR_018163). Only Caspr/Kv1.2 domains contained within YFP+ axons were included in the analysis. The percentage of typical and atypical Kv1.2 immunoreactive domains for each mouse was calculated based on the corresponding image area analyzed.

### Electron microscopy

Transmission EM was performed to assay the efficacy of 4-AP treatment by detecting ultrastructural pathology, including myelin integrity, of individual axons as in prior studies [7, 49, 54]. C57BL/6J male mice were perfused with fixative containing 2.5% glutaraldehyde and 4% PFA in 0.1 M phosphate buffer pH 7.4. The brains were dissected and post-fixed in the same fixative overnight. Sagittal 40 µm vibratome slices cut and osmicated to identify the CC region-of-interest (ROI), located above the lateral ventricles at the level of the crossing of the anterior commissure, for thin sectioning (~ 70 nm) followed by processing for EM. As noted above (see TBI Model), the midline crossing of the anterior commissure facilitates alignment with bregma (0 A-P) in the coronal plane, which is an area with a high proportion of myelinated axons as compared to more caudal regions of the mouse and rat corpus callosum [62, 70, 71].

### Quantification of axon and myelin damage

To assess the extent of ultrastructural pathology, axons were classified by an investigator blinded to surgical and treatment allocation. Twenty-to-fifteen representative images from each mouse were collected at 5,000 × magnification using a JEOL JEM-1011 transmission electron microscope (JEOL USA Inc., Peabody, MA) and an AMT XR50S-A digital camera (Advanced Microscopy Techniques, Woburn, MA) from which 10 images were randomly selected for analysis using the Excel RANDBETWEEN function in order to minimize investigator bias. A 17.8 µm × 13.4 µm counting frame was drawn over each image using Adobe Photoshop (RRID:SCR_014199) and axons touching the left and bottom margins of the counting frame were excluded from quantification. Damaged axons exhibiting abnormal mitochondrial swelling (> 50% of transverse axon area) were classified separately from axons exhibiting damage based on neurofilament compaction or vesicle/organelle accumulation. Axons large enough to typically be myelinated (diameter > 0.3 µm for adult mouse CC; [71] but lacking myelin were classified as de/unmyelinated. Experiments were conducted as cohorts of 2–3 mice for each of the four treatment groups, with 3 cohorts combined for the 3-day endpoint and 4 cohorts for the 7-day endpoint to generate the mouse numbers shown in Fig. 1A.

### Quantification of axon diameter, myelin thickness, and g-ratio

For each mouse, a grid measuring 3 µm × 3 µm was laid over one of the randomly selected EM images described above. All myelinated axons (range = 61–120 axons) contained within or touching the perimeter of every 3^rd^ grid cell within the counting frame were measured, and then the first 50 axons meeting the circularity criteria were included in the statistical analysis. The freehand selection tool in ImageJ was used to trace the axonal circumference generating automatic calculations of axon area and circularity. Axon diameter was calculated from axon area values using the formula 2 x √(area/π). Myelin thickness was determined based on the average of radial line measures across the myelin sheath taken at four equidistant points around the axon [49, 88]. Fiber diameter is 2 × myelin thickness + axon diameter. The g-ratio is the ratio of the axonal diameter to the fiber diameter. The fifty axons per mouse used in final calculations were approximately circular (having circularity > 0.6, where 1 = perfect circle) in order to avoid skewing of the data due to irregularly shaped axons or axons cut at a slant. In total, 350 axons were analyzed per sham animal group and 450 axons per TBI vehicle and TBI 4-AP groups.

### Ex Vivo electrophysiology of CC axons

Brain slices were used for ex vivo recordings to directly examine the electrophysiological properties of axons across the CC midline [50]. Following isoflurane anesthesia, brains were collected and submerged in ice-cold artificial cerebrospinal fluid (ACSF) with sucrose (in mM: 2 KCl, 1 CaCl_2_, 1.25 NaH_2_PO_4_, 2 MgSO_4_, 2 MgCl-6H_2_O, 26 NaHCO_3_, 10 D-glucose, 206 Sucrose), bubbled with a gas mixture of 95% O_2_/5% CO_2_. Coronal sections of 400 µm thickness were sliced using a Leica VT1200 vibratome (Leica Biosystems Inc., Buffalo Grove, IL) containing sucrose ACSF. Sections were transferred to a holding chamber filled with normal ACSF (in mM: 126 NaCl, 3 KCl, 2 CaCl_2_, 1.25 NaHPO_4_, 2 MgSO_4_, 2 MgCl-6H_2_O, 26 NaHCO_3_, 10 D-glucose; bubbled with 95% O_2_/5% CO_2_ mixture) at 36 °C for 30 min and then to room temperature for at least 1 h prior to recording. Individual slices were then transferred to a perfusion and recording chamber (Warner Instruments, Hamden, CT) on an upright Zeiss Examiner Z1and perfused with normal ACSF continuously bubbled with 95% O_2_/5% CO_2_ at a rate of 1–2 ml/min at room temperature. A bipolar tungsten electrode (10 k Ohm impedance) connected to a stimulus generator (S88K, Grass technologies, West Warwick, RI) was used for stimulation, while a borosilicate glass capillary (1 M Ohm resistance in the bath) was used as the recording electrode. Evoked field potentials in the CC were recorded from axons near the recording electrode placed approximately 1.5 ± 0.18 mm from the stimulating electrode.

### Compound action potential (CAP) and velocity measurements

The evoked CAP was captured using a MultiClamp 700B amplifier (Molecular Devices). Data was acquired with pClamp10 (pClamp; Molecular Devices, RRID:SCR_011323) and analyzed with Clampfit 10.4 (Molecular Devices) and OriginPro 2020b (OriginLab, Northampton, MA) software packages. Parameters of stimulation protocols were as follows: (a) Input–output recording: 50 µA to 700 µA in 50 µA steps, 0.2 ms duration; (b) Velocity measurement: three additional positions for the recording pipette were placed successively closer to the stimulus electrode (stimulus strength adjusted accordingly). OriginLab Pro software (RRID:SCR_014212) was used to determine: (a) CAP amplitude, measured from the peak of the CAP to a tangent line drawn over the projected CAP bases; (b) CAP width, computed at the CAP half-height; and (c) CAP peak to recovery, the latency from the peak to the return of the positive phase of the CAP. Conduction velocity was calculated based on the best-fit linear regression analysis of distance between electrodes (ranging from 0.4 to 1.8 mm) versus recording of the time interval between CAP waveform peaks. Conduction velocity and CAP measures were acquired for all mice included in the analysis. After the pre-determined interim data analysis at n = 6 mice per condition, 7 more mice were added per condition (see Exclusion section below). After completing recordings for CAP and conduction velocity, the brain slices of the added mice were also tested for more specific conduction parameters of refractoriness and strength-duration testing, which were not included in the pre-determined study design.

### Refractoriness and strength-duration analyses

For refractoriness testing, after a control single pulse was generated with 500 µA for 0.2 ms as stimulus, a paired pulse protocol [15, 61] was implemented in which pulses of the same strength and duration were separated by an inter-pulse interval (IPI) ranging from 1.5 to 8 ms in 0.5 ms increments. The paired pulse protocol was then followed by another control single pulse run. The average of the two control run amplitudes was used as 100% single pulse value. The ratio of the amplitude of each CAP component after the second paired pulse stimulation to the amplitude of the corresponding CAP amplitudes from the single pulse stimulation were plotted as a function of the interpulse interval. The 50% ratio of CAP_2_/CAP_1_ was used to compare refractoriness across groups. For the strength-duration measurement, after a control single pulse run (500 µA strength for 0.2 ms; considered 100% amplitude), recordings with increasing duration and adjusted stimulus strength were performed to elicit approximately 30% of the N1 and N2 amplitude of the control run. Duration steps were 50, 60, 80, 100, 120, 140, 160, 200, 300, 400, 500, and 600 ms. As the last step, a final control run was recorded with 0.2 ms duration and 500 µA strength to account for changes of recording conditions over time.

### Statistical analyses

Experimental data were analyzed and graphically represented using GraphPad Prism version 9.0.2 software (RRID:SCR_002798). Results are presented as mean ± standard error of the mean (SEM) unless otherwise noted. Two-group comparisons were made using unpaired Student’s *t*-test. One-way ANOVA tests were conducted when a single variable factor (injury) was tested in the analysis for the 4 animal groups (i.e., before 4-AP administration). Two-way ANOVA was used when interactions of both variable factors (injury and drug) were analyzed (see Tables 1, 2, 3, 4 for results). When the ANOVA test was found to be statistically significant, post hoc Tukey or Holm-Sidak tests were used to make pairwise comparisons between group means. For evaluating compound action potential and strength-duration data, a linear mixed effects model was used with injury and drug as random effects and current intensity as fixed effect, followed by post hoc Holm-Sidak multiple comparisons test. Multiple counts derived from sampling in a given mouse were averaged and treated as one data point in each quantitative figure, except for the g-ratio x axon diameter plot which depicts all axons measured in the analysis, and the CAP, strength-duration and refractoriness data whose data points depict group means. Effect size was estimated using Cohen’s *d* formula as the mean difference between two groups divided by the pooled standard deviation. Cohen defined *d* = 0.2,  0.5 and *d* ≥ 0.8 as small, medium and large effects, respectively [13]. All statistical tests were two-tailed and significance was set as p < 0.05.

**Table 1:** Study 1—Immunohistochemistry of axons in Thy1-YFP-16 mice (Figs. 2 and 3). Student’s *t*-test or two-way ANOVA results with Holm-Sidak post hoc test and Cohen’s *d* effect size

Figure 2- Outcome measures	t, df or F (DFn, DFd); *p*-value	Adjusted *p*-value	Cohen’s *d* effect size
YFP particles	t_12_ = 9.785; *p* < 0.0001 Interaction: F_1,10_ = 0.098, *p* = 0.7607 Sex: F_1,10_ = 0.022, *p* = 0.8852 Drug: F_1,10_ = 78.30, *p* < 0.0001	Males: TBI veh vs TBI 4-AP: *p* = 0.0006 Females: TBI veh vs TBI 4-AP: *p* = 0.0002 4-AP: TBI males vs TBI females: *p* = 0.9381	TBI veh vs TBI 4-AP: *d* = 5.231
% Area of YFP particles	t_12_ = 10.05; *p* < 0.0001 Interaction: F_1,10_ = 1.205, *p* = 0.2980 Sex: F_1,10_ = 0.00000275, *p* = 0.9987 Drug: F_1,10_ = 98.41, *p* < 0.0001	Males: TBI veh vs TBI 4-AP: *p* = 0.0005 Females: TBI veh vs TBI 4-AP: *p* < 0.0001 4-AP: TBI males vs TBI females: *p* = 0.7028	TBI veh vs TBI 4-AP: *d* = 5.370

Figure 3- Outcome measures	F (DFn, DFd); *p*-value	Adjusted *p*-value	Cohen’s *d* effect size
Caspr+ heminodes	Interaction: F_1,12_ = 17.21, *p* = 0.0013 Injury: F_1,12_ = 61.00, *p* < 0.0001 Drug: F_1,12_ = 16.80, *p* = 0.0015	sham veh vs TBI veh: *p* < 0.0001 sham 4-AP vs TBI 4-AP: ns, *p* = 0.0651 TBI veh vs TBI 4-AP: *p* = 0.0003	sham veh vs TBI veh: *d* = 5.591 sham 4-AP vs TBI 4-AP: *d* = 1.917 TBI veh vs TBI 4-AP: *d* = 3.713
Caspr length difference	Interaction: F_1,12_ = 15.66, *p* = 0.0019 Injury: F_1,12_ = 31.46, *p* = 0.0001 Drug: F_1,12_ = 15.66, *p* = 0.0019	sham veh vs TBI veh: *p* = 0.0001 sham 4-AP vs TBI 4-AP: ns, *p* = 0.6040 TBI veh vs TBI 4-AP: *p* = 0.0005	sham veh vs TBI veh: *d* = 5.340 sham 4-AP vs TBI 4-AP: *d* = 0.754 TBI veh vs TBI 4-AP: *d* = 3.302
% Atypical Kv1.2 domains	Interaction: F_1,12_ = 14.47, *p* = 0.0025 Injury: F_1,12_ = 293.7, *p* < 0.0001 Drug: F_1,12_ = 7.281, *p* = 0.0194	sham veh vs TBI veh: *p* < 0.0001 sham 4-AP vs TBI 4-AP: *p* < 0.0001 TBI veh vs TBI 4-AP: *p* = 0.0012	sham veh vs TBI veh: *d* = 11.136 sham 4-AP vs TBI 4-AP: *d* = 6.312 TBI veh vs TBI 4-AP: *d* = 5.511

*veh* Saline vehicle control; *ns* Non-significant (*p* > 0.05). Sham veh vs sham 4-AP ns for each measure (*p* > 0.4 or higher). Veh TBI males vs veh TBI females ns for each measure (*p* > 0.7 or higher)

**Table 2:** Study 2 — Electron microscopy identification of axon pathological features (Fig. 4). Two -way ANOVA results with Holm-Sidak post hoc test and Cohen’s *d* effect size

Outcome measure	F (DFn, DFd); *p*-value	Adjusted *p*-value	Cohen’s *d* effect size
3-day Intact myelinated	Interaction: F_1,31_ = 0.5948, *p* = 0.4464 Injury: F_1,31_ = 9.045, *p* = 0.0052 Drug: F_1,31_ = 1.863, *p* = 0.1821	sham veh vs TBI veh: ns, *p* = 0.0527 sham 4-AP vs TBI 4-AP: ns, *p* = 0.4255 TBI veh vs TBI 4-AP: ns, *p* = 0.4255	sham veh vs TBI veh: *d* = 1.212 sham 4-AP vs TBI 4-AP: *d* = 0.799 TBI veh vs TBI 4-AP: *d* = 0.691
3-day Axons with swollen mitochondria	Interaction: F_1,31_ = 0.07855, *p* = 0.7811 Injury: F_1,31_ = 63.85, *p* < 0.0001 Drug: F_1,31_ = 0.04218, *p* = 8386	sham veh vs TBI veh: *p* < 0.0001 sham 4-AP vs TBI 4-AP: *p* < 0.0001 TBI veh vs TBI 4-AP: ns, *p* = 0.9310	sham veh vs TBI veh: *d* = 2.955 sham 4-AP vs TBI 4-AP: *d* = 2.398 TBI veh vs TBI 4-AP: *d* = 0.115
3-day Damaged axons	Interaction: F_1,31_ = 1.236, *p* = 0.2749 Injury: F_1,31_ = 205.8, p < 0.0001 Drug: F_1,31_ = 1.295, p = 0.2639	sham veh vs TBI veh: *p* < 0.0001 sham 4-AP vs TBI 4-AP: *p* < 0.0001 TBI veh vs TBI 4-AP: ns, *p* = 0.2383	sham veh vs TBI veh: *d* = 4.232 sham 4-AP vs TBI 4-AP: *d* = 6.550 TBI veh vs TBI 4-AP: *d* = 0.539
3-day De/unmyelinated axons	Interaction: F_1,31_ = 3.382, *p* = 0.0755 Injury: F_1,31_ = 49.34, p < 0.0001 Drug: F_1,31_ = 3.032, p = 0.0916	sham veh vs TBI veh: *p* < 0.0001 sham 4-AP vs TBI 4-AP: *p* = 0.0035 TBI veh vs TBI 4-AP: *p* = 0.0360	sham veh vs TBI veh: *d* = 3.144 sham 4-AP vs TBI 4-AP: *d* = 1.677 TBI veh vs TBI 4-AP: *d* = 1.140
7-day Intact myelinated	Interaction: F_1,35_ = 4.099, *p* = 0.0506 Injury: F_1,35_ = 14.27, p = 0.0006 Drug: F_1,35_ = 0.3801, p = 0.5416	sham veh vs TBI veh: *p* = 0.0012 sham 4-AP vs TBI 4-AP: ns, *p* = 0.4061 TBI veh vs TBI 4-AP: ns, *p* = 0.1868	sham veh vs TBI veh: *d* = 1.707 sham 4-AP vs TBI 4-AP: *d* = 0.636 TBI veh vs TBI 4-AP: *d* = 0.772
7-day Axons with swollen mitochondria	Interaction: F_1,35_ = 5.852, *p* = 0.0209 Injury: F_1,35_ = 203.3, p < 0.0001 Drug: F_1,35_ = 8.069, p = 0.0075	sham veh vs TBI veh: *p* < 0.0001 sham 4-AP vs TBI 4-AP: *p* < 0.0001 TBI veh vs TBI 4-AP: *p* = 0.0012	sham veh vs TBI veh: *d* = 4.879 sham 4-AP vs TBI 4-AP: *d* = 4.386 TBI veh vs TBI 4-AP: *d* = 1.247
7-day Damaged axons	Interaction: F_1,35_ = 20.73, *p* < 0.0001 Injury: F_1,35_ = 170.1, p < 0.0001 Drug: F_1,35_ = 20.73, p < 0.0001	sham veh vs TBI veh: *p* < 0.0001 sham 4-AP vs TBI 4-AP: *p* < 0.0001 TBI veh vs TBI 4-AP: *p* < 0.0001	sham veh vs TBI veh: *d* = 4.235 sham 4-AP vs TBI 4-AP: *d* = 6.596 TBI veh vs TBI 4-AP: *d* = 2.094
7-day De/unmyelinated axons	Interaction: F_1,35_ = 16.83, *p* = 0.0002 Injury: F_1,35_ = 32.71, p < 0.0001 Drug: F_1,35_ = 15.33, p = 0.0004	sham veh vs TBI veh: *p* < 0.0001 sham 4-AP vs TBI 4-AP: ns, *p* = 0.4963 TBI veh vs TBI 4-AP: *p* < 0.0001	sham veh vs TBI veh: *d* = 2.618 sham 4-AP vs TBI 4-AP: *d* = 0.723 TBI veh vs TBI 4-AP: *d* = 1.973

*veh* Saline vehicle control; ns: non-significant (*p* > 0.05). Sham veh vs sham 4-AP ns for each measure (*p* > 0.4 or higher)

**Table 3:** Study 2—Electron microscopy analysis of fiber morphometry (Fig. 5). Two -way ANOVA results with Holm-Sidak post hoc test and Cohen’s *d* effect size

Outcome measure	F (DFn, DFd); *p*-value	Adjusted *p*-value	Cohen’s d effect size
Axon diameter	Interaction: F_1,28_ = 0.1513, *p* = 0.7002 Injury: F_1,28_ = 11.08, *p* = 0.0025 Drug: F_1,28_ = 0.01632, *p* = 0.8993	sham veh vs TBI veh: ns, *p* = 0.1343 sham 4-AP vs TBI 4-AP: ns, *p* = 0.0798 TBI veh vs TBI 4-AP: ns, *p* = 0.9287	sham veh vs TBI veh: *d* = 0.959 sham 4-AP vs TBI 4-AP: *d* = 1.380 TBI veh vs TBI 4-AP: *d* = 0.115
Myelin thickness	Interaction: F_1,28_ = 1.159, *p* = 0.2908 Injury: F_1,28_ = 106.7, *p* < 0.0001 Drug: F_1,28_ = 0.09095, *p* = 0.7652	sham veh vs TBI veh: *p* < 0.0001 sham 4-AP vs TBI 4-AP: *p* < 0.0001 TBI veh vs TBI 4-AP: ns, p = 0.5189	sham veh vs TBI veh: *d* = 3.081 sham 4-AP vs TBI 4-AP: *d* = 4.219 TBI veh vs TBI 4-AP: *d* = 0.563

*veh* saline vehicle control; ns: non-significant (*p* > 0.05). Sham veh vs sham 4-AP ns for each measure (*p* > 0.6 or higher)

**Table 4:** Study 3—Electrophysiology of compound action potential (CAP) waveforms (Fig. 6). Two-way ANOVA results with Holm-Sidak post hoc test and Cohen’s* d* effect size

Outcome measure	F (DFn, DFd); *p*-value	Adjusted *p*-value	Cohen’s d effect size
N1 conduction velocity	Interaction: F_1,43_ = 0.05412, *p* = 0.8171 Injury: F_1,43_ = 26.20, *p* < 0.0001 Drug: F_1,43_ = 1.056, *p* = 0.3098	sham veh vs TBI veh: *p* = 0.0067 sham 4-AP vs TBI 4-AP: *p* = 0.0016 TBI veh vs TBI 4-AP: ns, *p* = 0.6182	sham veh vs TBI veh: *d* = 1.646 sham 4-AP vs TBI 4-AP: *d* = 1.405 TBI veh vs TBI 4-AP: *d* = 0.286
N2 conduction velocity	Interaction: F_1,43_ = 0.2463, *p* = 0.6222 Injury: F_1,43_ = 6.691, *p* = 0.0132 Drug: F_1,43_ = 0.9846, *p* = 0.3266	sham veh vs TBI veh: ns, *p* = 0.1860 sham 4-AP vs TBI 4-AP: ns, *p* = 0.4377 TBI veh vs TBI 4-AP: ns, *p* = 0.5970	sham veh vs TBI veh: *d* = 0.945 sham 4-AP vs TBI 4-AP: *d* = 0.582 TBI veh vs TBI 4-AP: *d* = 0.577
N2 width at half-maximum	Interaction: F_1,43_ = 0.1562, *p* = 0.6947 Injury: F_1,43_ = 7.827, *p* = 0.0077 Drug: F_1,43_ = 0.1402, *p* = 0.7099	sham veh vs TBI veh: ns, *p* = 0.1601 sham 4-AP vs TBI 4-AP: ns, *p* = 0.3032 TBI veh vs TBI 4-AP: ns, *p* = 0.9883	sham veh vs TBI veh: *d* = 1.003 sham 4-AP vs TBI 4-AP: *d* = 0.675 TBI veh vs TBI 4-AP: *d* = 0.008
N2 peak to recovery	Interaction: F_1,43_ = 2.797, *p* = 0.1017 Injury: F_1,43_ = 5.211, *p* = 0.0275 Drug: F_1,43_ = 0.08605, *p* = 0.7707	sham veh vs TBI veh: ns, *p* = 0.0560 sham 4-AP vs TBI 4-AP: ns, *p* = 0.6579 TBI veh vs TBI 4-AP: ns, *p* = 0.5517	sham veh vs TBI veh: *d* = 1.240 sham 4-AP vs TBI 4-AP: *d* = 0.171 TBI veh vs TBI 4-AP: *d* = 0.365

*veh* Saline vehicle control; ns: non-significant (*p* > 0.05). Sham veh vs sham 4-AP ns for each measure (*p* > 0.5 or higher)

### Study exclusion criteria

Predetermined exclusion criteria included: (a) 10% body weight loss at any point during experimentation (n = 0); (b) mice exhibiting an incongruous righting reflex (< 2 min for TBI animals; > 2 min for sham), a fractured, depressed skull or bleeding after impact, and/or signs of pain or distress at any point during the experiment (n = 2, fractured skull, Study 2 – Fig. 1A); (c) failure to meet technical quality standards for brain slices (n = 4, anatomical region not intact after cutting). In addition, one mouse from Study 3 – Electrophysiology had to be excluded due to building site closure in response to the COVID-19 pandemic.

## Results

### Characterization of study group variables and 4-AP serum levels

The effect of 4-AP on white matter injury in the acute phase of TBI was examined in the CC using a well characterized closed head model [7, 50, 54]. A pre-determined randomized, controlled study design, with blinding, was used to allocate mice to groups in each of three separate studies (Fig. 1A). To evaluate comparability among the allocated groups, potential confounding variables were analyzed across the experiments. TBI mice exhibited a significantly longer righting time compared to sham controls (Fig. 1B), which confirms the injury effect on this surrogate measure of altered consciousness. The similar delay in the righting reflex of TBI mice allocated to vehicle and 4-AP groups indicates comparable response to injury prior to treatment initiation at 24 h after the TBI or sham procedure (Fig. 1B). The mean body weight among all groups of mice remained stable relative to pre-surgical measurements (Fig. 1C), which shows that neither TBI nor 4-AP treatment have detrimental effects on overall health. Furthermore, mice did not exhibit overt signs of adverse side effects such as altered grooming and posture, hyperactivity, tremors, convulsions, or cardiac arrest. Based on these observations, the low clinically relevant 4-AP dose of 0.5 mg/kg used in this study did not produce poor health or behavioral evidence of convulsant effects. 4-AP acts as a convulsant in adult mice at much higher bolus doses of over 5 mg/kg (ED_50_ of 10.9 mg/kg; [82].

Treatment with 4-AP began 24 h after the sham or TBI surgeries in order to provide a clinically reasonable time to initiation of treatment, as in previous studies of 4-AP treatment on acute peripheral nerve injury [74]. To determine the 4-AP circulating levels following the 0.5 mg/kg bolus injections, 4-AP was measured in cardiac serum samples drawn prior to perfusion at approximately 30–60 min after the last 4-AP or vehicle injection (Fig. 1D). Separate experiments in a cohort of 12 mice (n = 3/group) had delayed blood sample collection (2.75–7.25 h) after the last injection and found that the 4-AP levels in serum dropped to below detectable levels (data not shown), in agreement with a short (~ 2 h) half-life in rodents [11, 66]. This data demonstrates that the low 4-AP dosage used in this investigation was near or below the clinically acceptable serum dose range of 20–100 ng/ml and overt signs of unacceptable adverse side effects were not observed.

### Acute 4-AP treatment reduces axon damage in the CC after TBI

**Fig. 2:**
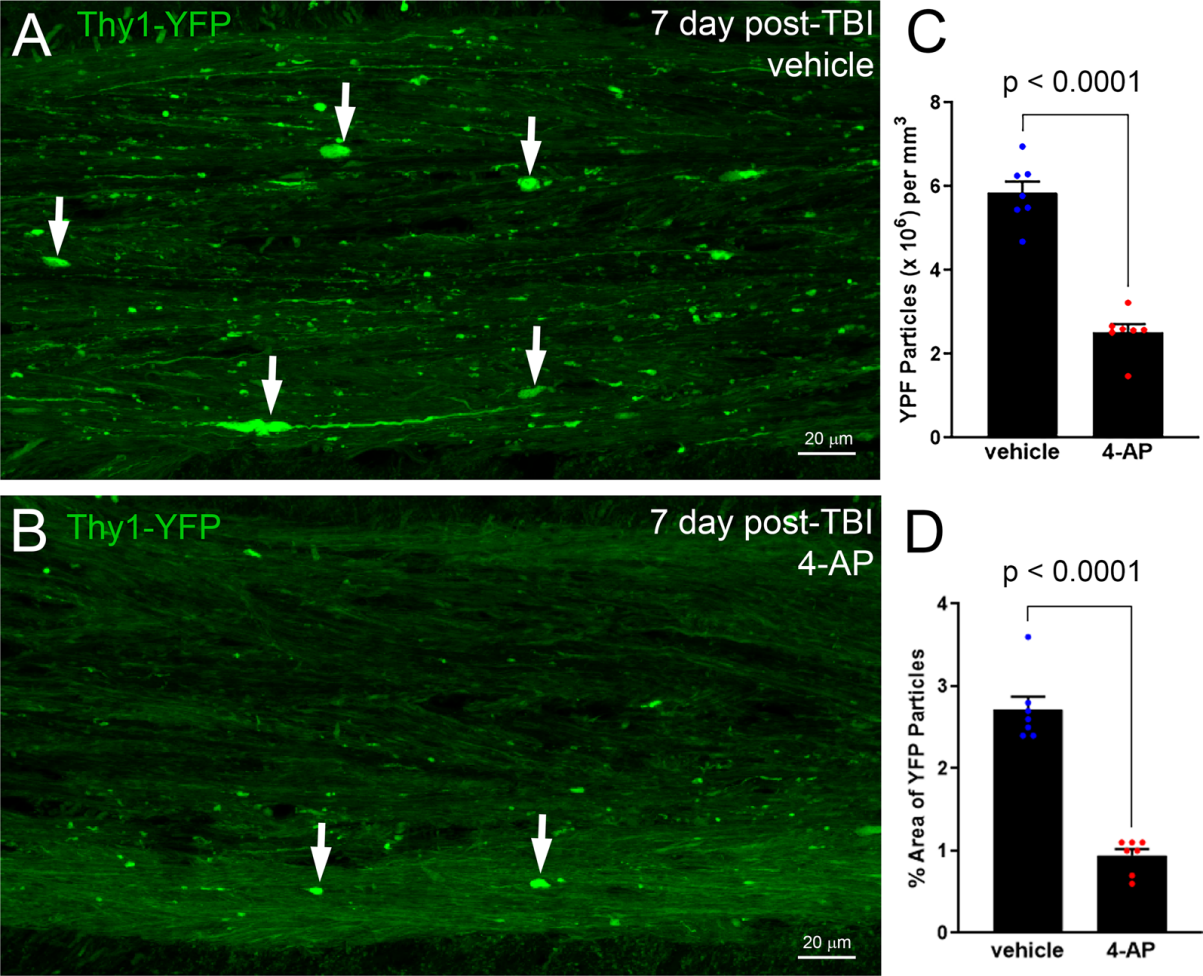
4-AP treatment shows potent axon protection using YFP-filled swellings to screen for damaged axons. **A–B** Thy1-YFP-16 mice were given vehicle (**A**) or 4-AP (**B**) treatment on days 1–7 after TBI. Confocal images of axons in the corpus callosum illustrate YFP (green) fluorescence accumulation in axonal swellings (white arrows), which is a sensitive indicator of axon damage. Adjacent YFP-positive axons have YFP diffusely distributed along normal appearing axons. **C–D** ImageJ particle analysis of YFP accumulation in axonal swellings. 4-AP treatment reduced the density (**C**) and the percent area occupied by axon swellings (**D**). Bars represent mean ± SEM with an individual data point shown for each mouse. Unpaired Student’s *t*-tests. See Fig. 1 for mouse sample numbers and Table 1 for statistical details

To determine the effect of 4-AP administration on axon damage after TBI, we developed a semi-automated screening assay using Thy1-YFP-16 reporter mice (Study 1, Fig. 1A). In these mice, yellow fluorescence protein (YFP) is expressed in a broad spectrum of projection neurons crossing through the CC [30]. In healthy adult mice, fluorescence is evenly distributed throughout the cytoplasm of axons expressing the YFP transgene. TBI produced dispersed but widespread axonal injury that was visible as robust YFP accumulations in axonal swellings throughout the CC of TBI mice in the vehicle group (Fig. 2A). Only nominal levels of small YFP+ axonal accumulations were detected in sham mice (data not shown, and as published in [50]). Acute 4-AP treatment on days 1–7 post-TBI dramatically reduced axonal YFP+ swellings and accumulations in TBI mice (Fig. 2B).

Matched regions of the CC were quantified at 7 days after TBI in mice treated with either 4-AP or vehicle, and indicated that 4-AP treatment mitigated axonal damage as revealed by YFP detection. This semi-automated approach enabled sampling of a relatively large region of the CC and takes advantage of YFP detection of subtle damage in swellings through to formation of terminal end bulbs at points of axon disconnection [6, 35, 78]. 4-AP treatment significantly decreased the number of YFP-filled swellings (Fig. 2C). The overall proportion of the CC area occupied by YFP+axonal swellings was also significantly less in 4-AP treated mice (Fig. 2D). A statistically significant difference was not found between male and female mice of either treatment group (Table 1). Therefore, subsequent studies used only male mice. Taken together, these results yielded large effect sizes for axon damage reduction following 4-AP treatment with YFP accumulations quantified based on the number (*d* = 5.231) or percent area occupied (*d* = 5.370). This screening experiment provided compelling support for more extensive analyses with continuing to initiate treatment one day after TBI and delivering 4-AP as twice daily (0.5 mg/kg) injections.

### Acute 4-AP treatment reduces disorganization of nodal regions after TBI

**Fig. 3:**
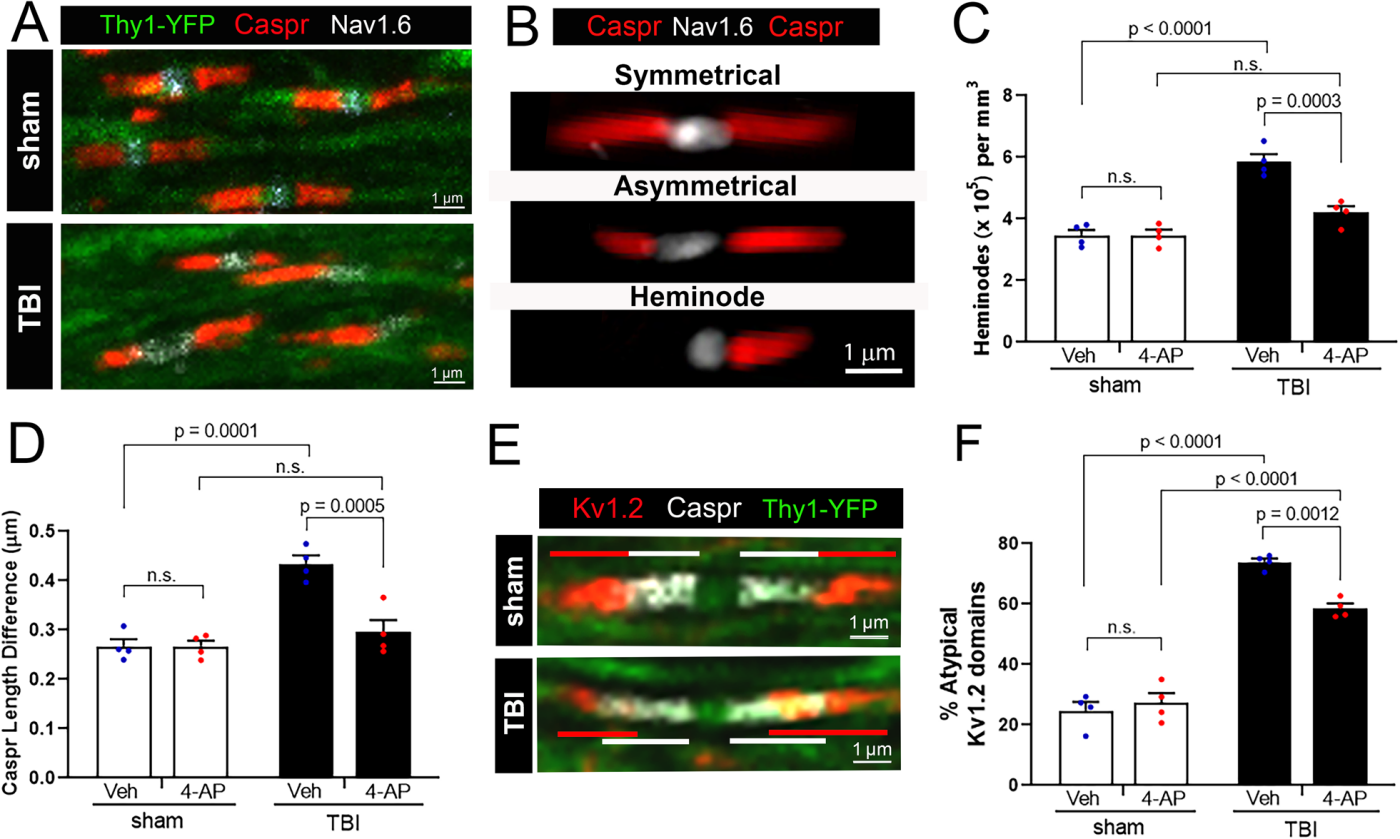
4-AP treatment improves the molecular organization of excitable axonal domains that are disrupted by TBI. Confocal imaging analysis of node of Ranvier complexes in individual corpus callosum axons of Thy1-YFP-16 mice at 7 days post-TBI or sham procedures. (**A**) Immunostaining along YFP-labeled axons (green) detected clustering of voltage-gated sodium channels (Nav1.6, white) at the node and Caspr (red) in the paranode region, where myelin attaches to the axon. **B-D** TBI disrupted paranode domain organization, which was normalized by 4-AP treatment. (**B**) “Symmetrical” Caspr paranodes exhibit paired Caspr bands of approximately symmetrical length, while “Asymmetrical” paranodes exhibit uneven Caspr domain lengths. Single Caspr paranodes with a missing domain counterpart on the opposite side of a node were classified as “Heminodes”. **C** TBI increased the number of Caspr heminodes while acute 4-AP treatment post-TBI resulted in heminode numbers similar to sham levels. **D** TBI increased the asymmetry of Caspr domains in TBI mice compared to sham. 4-AP treatment significantly improved paranode organization following TBI. **E–F** Kv1.2 channel (red) and Caspr (white) immunostaining along individual YFP (green) axons in single confocal optical slices. **E** In injured mice, Kv1.2 channels mislocalize from the juxtaparanode domain into Caspr-labeled paranode domain. **F** Quantification of atypical Kv1.2 domains that overlapped with Caspr domains and/or were asymmetrical in length. Acute 4-AP treatment reduced the percentage of atypical Kv1.2 domains after TBI, but the distribution patterns of Kv1.2 channels was not fully normalized to sham levels. **C, D, F** Bars represent mean ± SEM with an individual data point shown for each mouse. Two-way ANOVA for main effect of injury or drug with Holm-Sidak’s multiple comparisons test for significance of post-hoc pair effects. See Fig. 1 for mouse sample numbers and Table 1 for statistical details

Axon domains associated with the node of Ranvier that are essential for rapid conduction of nerve impulses were investigated in Thy1-YFP-16 mice to explore potential 4-AP effects related to this aspect of axonal damage (Fig. 3). We have previously shown TBI disrupts axonal paranodes that flank nodes of Ranvier [50]. Coronal brain sections through the CC were immunolabeled for contactin-associated protein (Caspr) and the 1.6 isoform of voltage-gated sodium channels (Nav1.6). In myelinated axons, Caspr proteins are localized in axonal paranodes, which are sites of myelin attachment that flank nodes of Ranvier containing Nav1.6 channels (Fig. 3A). TBI disrupted axon-myelin interactions at the paranodes based on the altered distribution of Caspr immunoreactivity at paranodes (Fig. 3A). While most paired Caspr domains appear symmetrical, i.e. similar in length on both sides of a given node, others were asymmetrical (Fig. 3B). Heminodes were also present as single (unpaired) Caspr immunoreactive domains adjacent to Nav1.6 labeled nodes along YFP-expressing axons (Fig. 3B).

We found that 4-AP treatment protected against TBI-induced changes in Caspr paranode domains. In the vehicle conditions, TBI increased Caspr heminodes and asymmetry in the paranodes as compared to sham mice (Fig. 3C, D). 4-AP treatment led to a substantial decrease in post-TBI heminodes and asymmetrical paranodes, with levels comparable to sham mice at the 7-day study endpoint (Fig. 3C, D). The nodal gap between Caspr pairs was not significantly different between groups (data not shown).

Immunolabeling Thy1-YFP-16 sections for Kv1.2 channel to examine channel distribution revealed that 4-AP treatment significantly improved Kv1.2 localization. Axonal Kv1.2 voltage-gated potassium channels, which are a potential therapeutic target of 4-AP, are localized in juxtaparanode regions under the myelin sheath in healthy myelinated axons. Caspr containing paranodes segregate the ionic currents of potassium channels in juxtaparanodes from the sodium channels in the node of Ranvier, which is critical for action potential propagation. In healthy axons, Caspr paranode domains and adjacent Kv1.2 juxtaparanode domains are clearly separated with minimal overlap (see sham example in Fig. 3E). TBI caused a loss of domain boundaries, with Caspr and Kv1.2 domains markedly overlapping with each other (see TBI example in Fig. 3E). In vehicle controls, TBI caused disordered atypical Kv1.2 immunolabeling patterns (Fig. 3F). Specifically, TBI increased the proportion of axons with Kv1.2 overlapping into paranodal (Caspr+) regions, asymmetry between paired Kv1.2 domains, and/or Kv1.2 dispersed away from the nodal region. After TBI, 4-AP treatment significantly improved Kv1.2 localization as compared to the vehicle condition (Fig. 3F). However, atypical Kv1.2 domains in TBI mice remained significantly above sham levels. This analysis shows significant improvement, but not full restoration, of the complex molecular domains of nodal regions during the first week post-TBI with 4-AP treatment.

### Acute 4-AP treatment reduces axon damage and myelin loss during the first week post-TBI

While YFP fluorescence sensitively detects axon swellings as an indicator of damage, electron microscopy is the gold standard approach to examine distinct features of pathology in axons and their ensheathing myelin. Electron microscopy was used to evaluate 4-AP effects on axon damage and demyelination in the CC after TBI (Study 2, Fig. 1A). Studies were conducted with 4-AP (0.5 mg/kg; i.p.) or saline vehicle injections initiated at 24 h after TBI or sham procedures and continued twice daily until perfusion on day 3 or 7. The 3-day time point was chosen to allow comparison with our previous ultrastructural characterization of axon and myelin pathology in this concussive TBI model [49, 54]. The 7-day time point aligns with the analysis in Thy1-YFP-16 mice for in-depth interpretation of the axon damage and nodal region pathology (Figs. 2, 3).

**Fig. 4:**
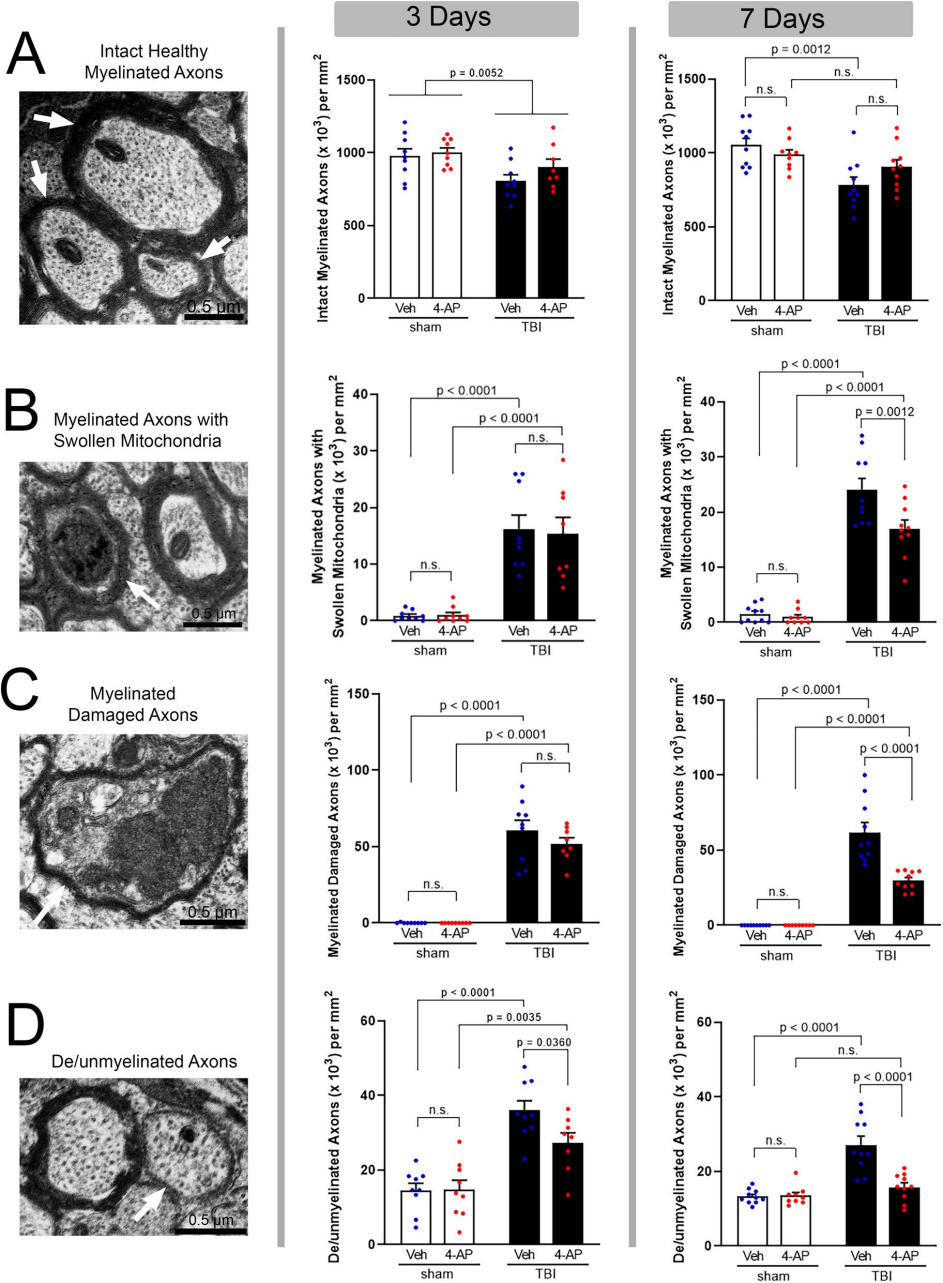
4-AP benefit requires continued treatment during the first week post-TBI to protect against multiple features of axon damage. Electron microscopy imaging of axon and myelin ultrastructural features (left) with classification of axon pathology at 3 days (middle) or 7 days (right) after TBI or sham procedure. ***Left panels:*** Examples of axon and myelin ultrastructural features. **A** Intact healthy myelinated axons (arrows). **B** Swollen mitochondrion (i.e., occupying over 50% of the axon cross section; arrow) in contrast to the typical mitochondrion in the adjacent axon. **C** Damaged myelinated axon (arrow) exhibiting condensed cytoplasm and vesicle/organelle accumulation. **D** Demyelinated axon (arrow) lacking a myelin sheath but structurally intact with diameter > 0.3 µm, which is typically myelinated. ***Middle Panels:*** At the 3-day time point, TBI significantly reduced the number of intact myelinated axons (**A**; only TBI main effect significant). TBI significantly increased myelinated axons with swollen mitochondria (**B**) or axon damage (**C**), and induced demyelination of additional axons (**D**) in both vehicle and 4-AP groups at 3 days. 4-AP treatment on days 1–3 did not have a significant benefit as compared to vehicle (**B**) or the number of damaged (**C**) and demyelinated (**D**) axons post-TBI. ***Right Panels:*** With 4-AP treatment extended to 7 days, the number of intact myelinated axons post-TBI was statistically similar to sham levels (**A**). More specifically, 4-AP treatment significantly reduced axons exhibiting mitochondrial swelling (**B**), axon damage (**C**), or TBI-induced demyelination (**D**). **A**–**D** Bars represent mean ± SEM with an individual data point shown for each mouse. Two-way ANOVA for main effects of injury or drug with Holm-Sidak’s multiple comparisons test of significance for post-hoc pair effects. See Fig. 1 for mouse sample numbers and Table 2 for statistical details

Quantitative analysis showed that TBI reduced the healthy-appearing, i.e. intact, myelinated axons (Fig. 4A). In agreement with this finding, in the vehicle condition, TBI resulted in significant pathology in myelinated axons that was identified based on specific ultrastructural features. Healthy-appearing axons exhibiting only swollen mitochondrial profiles were increased after TBI (Fig. 4B) and may represent an early state of axonal injury [3, 57]. TBI resulted in damaged axons that exhibited condensed cytoplasm, with compacted cytoskeletal structure, and vesicle or organelle accumulations (Fig. 4C). Axons with diameters greater than 0.3 µm, which are typically large enough to be myelinated in adult mouse CC [71], that lacked myelin were classified as de/unmyelinated axons. TBI-induced demyelination was indicated by de/unmyelinated values above sham levels (Fig. 4D).

4-AP treatment through 7 days (Fig. 4, right column) showed significant benefits on axon and myelin pathology, which were not evident at 3 days post-TBI (Fig. 4, middle column). At the 7 day time point, as compared to vehicle, 4-AP significantly reduced TBI pathology for axons with swollen mitochondria by 29.5% (Fig. 4B), damaged axons by 51.7% (Fig. 4C), and de/unmyelinated axons by 41.7% (Fig. 4D). In the case of axons with swollen mitochondria, 4-AP treatment appeared to attenuate further deterioration in the axons. In the case of damaged and de/unmyelinated axons, 4-AP treatment appeared to enhance recovery between the 3 and 7 day time points. Each of the three pathological features was significantly reduced with the longer duration of 4-AP treatment compared to vehicle-treated TBI mice, and de/unmyelinated axon values recovered to sham levels. These results demonstrate that 4-AP reduces axon damage and demyelination during a therapeutic window from one day after the mechanical injury through the first week post-TBI.

**Fig. 5:**
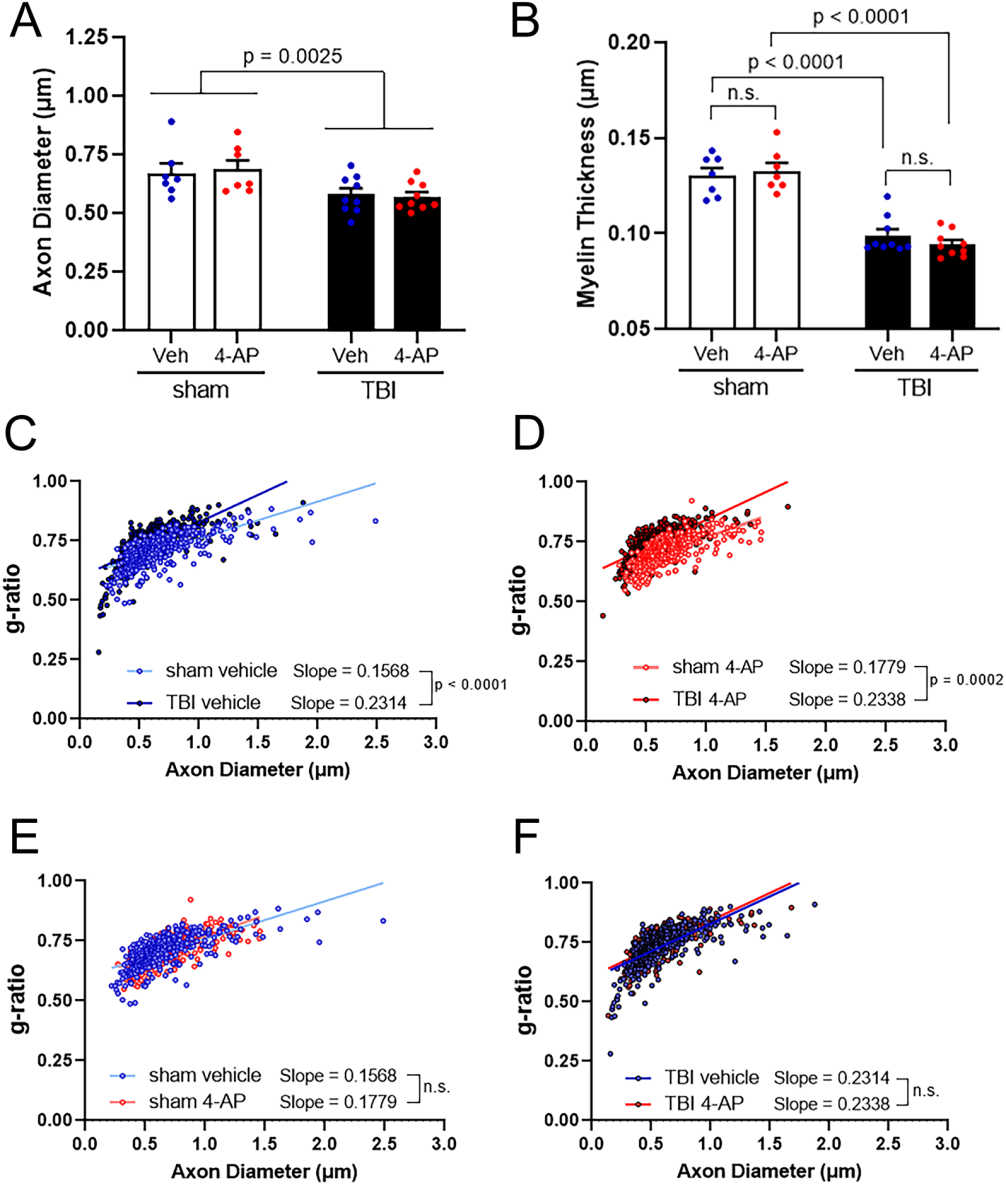
TBI reduces axon diameter and myelin thickness in 4-AP and vehicle conditions. Morphological analysis of intact myelinated axons in the corpus callosum of mice treated with 4-AP or vehicle on days 1–7 post-TBI or sham condition. **A** A main effect of TBI results in atrophic axons regardless of vehicle or 4-AP treatment. **B** Intact axons have thinner myelin after TBI in both vehicle and 4-AP treated mice. **C**–**F** Scatter plots display axon diameter against g-ratio (inner axonal diameter divided by total outer diameter), since appropriate myelin thickness is related to the diameter of a given axon. The slope of the linear regression was steeper for TBI versus sham mice. This indicates myelin thinning in proportion to axon diameter after TBI. This relationship was not influenced by 4-AP treatment as compared to vehicle (E, sham *p* = 0.1362; F, TBI *p* = 0.8818). **A**–**B** Two-way ANOVA for main effect of injury or drug with Holm-Sidak’s multiple comparisons test of significance for post-hoc pair effects. Bars represent mean ± SEM with an individual data point shown for each mouse. **C**–**F** Linear regression analysis. Each circle represents a measured axon (50 axons/mouse). See Fig. 1 for mouse sample numbers and Table 3 for statistical details

The EM images of CC axons at the 7 day time point were further examined to quantify axon diameter and myelin thickness, which can both influence axon conduction properties (Fig. 5). The mean diameter of myelinated axons decreased significantly in TBI conditions compared to sham mice (Fig. 5A). TBI also significantly reduced the myelin sheath thickness in both vehicle and 4-AP treated mice compared to sham controls (Fig. 5B). A specific effect of 4-AP treatment was not found for either axon diameter or myelin thickness. In adult white matter, myelin thickness varies with axon diameter. This relationship was examined using the g-ratio, which is the ratio of the axon diameter to the diameter of the myelinated axon fiber. Comparison of the g-ratio relative to axon diameter showed that myelin was thinner due to TBI as compared to sham, which was found in both 4-AP and vehicle groups (Fig. 5C, D). The g-ratio was not changed by 4-AP treatment in sham or TBI groups (Fig. 5E, F).

### Acute 4-AP treatment does not ameliorate functional deficits of CC axon populations within the first week after TBI

**Fig. 6:**
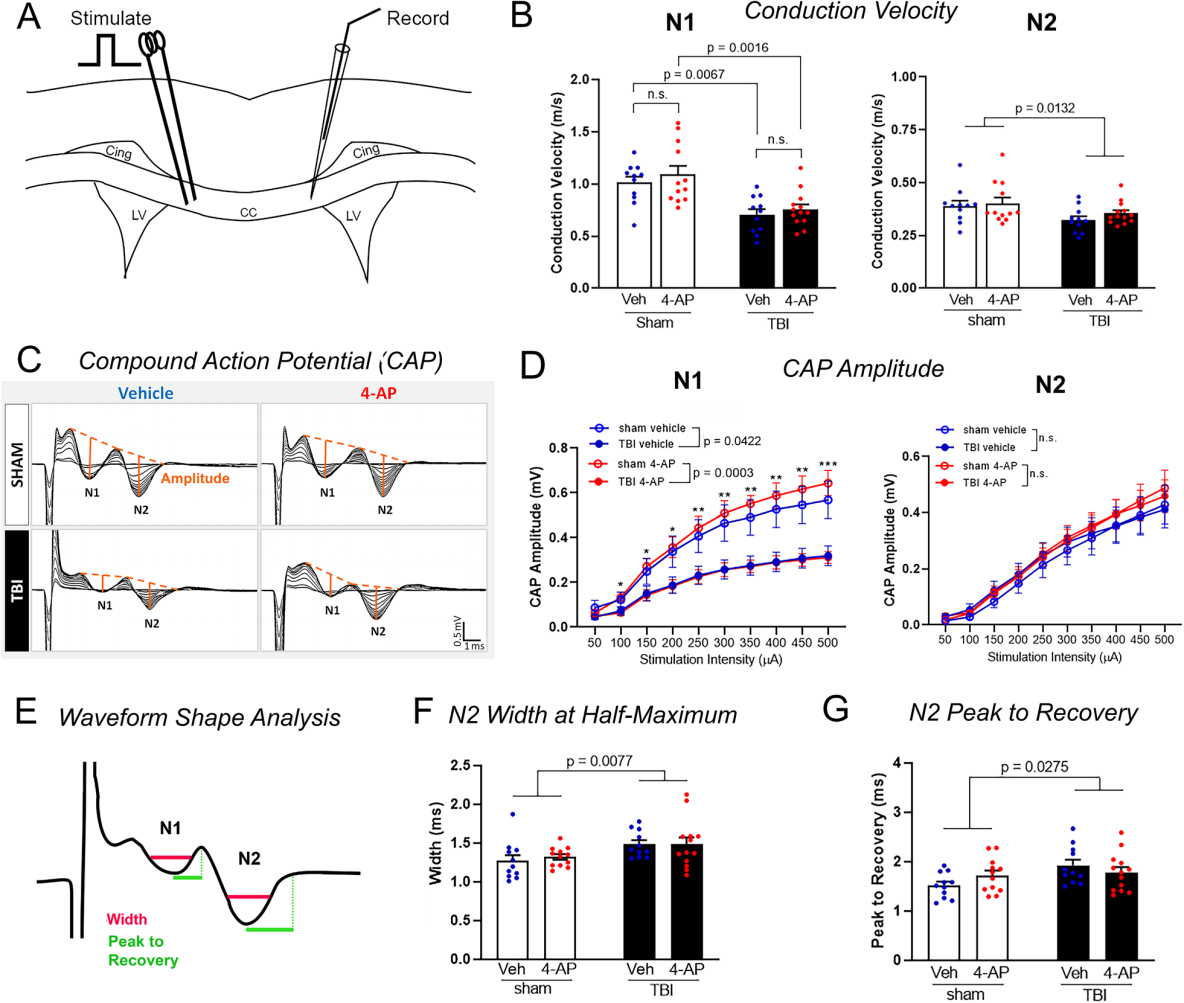
TBI slows the axon compound action potential velocity and amplitude. Electrophysiology recordings were used to directly test the function of axons in the corpus callosum. **A** Schematic of the position of the stimulating and recording electrodes for measurements of axon compound action potentials (CAP) and conduction velocity in ex vivo brain slices. **B** TBI reduced the speed of action potential conduction in both faster N1 wave comprised of myelinated axons and slower N2 wave comprised of unmyelinated and potentially demyelinated axons. The 7-day 4-AP treatment regimen did not restore this velocity deficit. **C** Representative input–output traces show the evoked N1 and N2 CAP waveforms with stimulus intensity pulses ranging from 50 to 500 µA (50 µA increments). Orange lines from CAP peaks to their projected bases indicate the amplitude of the response of fibers at a given stimulus intensity. **D** CAP amplitude analysis revealed a main injury effect on myelinated axons based on the reduced amplitude of only the N1 wave. N1 CAP amplitude difference was significant with post-hoc comparison for the sham versus TBI mice with 4-AP treatment. **E** Schematic of complementary spike waveform parameters. The width is dependent on the conduction velocity distribution among contributing axons. The time from peak to recovery represents the time for membrane repolarization among the slowest conducting axons comprising each waveform. The CAP width (**F**) and time from peak to recovery (**G**) indicated that TBI prolonged the recovery of the N2 waveform in both vehicle and 4-AP conditions. The N1 wave shape parameters were not altered by TBI or 4-AP treatment (data not shown). Abbreviations: Cing, cingulum; CC, corpus callosum; LV, lateral ventricles. **B**, **F**, **G** Bars represent mean ± SEM with individual data points for each mouse (n = 11–13 animals per group). **D** Linear effects model statistical analysis and Holm-Sidak’s multiple comparison test (*p < 0.05, **p < 0.01, ***p < 0.001). Two-way ANOVA for main effect of injury or drug with Holm-Sidak’s multiple comparisons test for post-hoc comparison of pair effects. See Fig. 1 for mouse sample numbers and Table 4 for statistical details

To evaluate axon function relative to TBI and systemic 4-AP administration, ex vivo studies of brain slices were used to directly measure signal conduction properties of CC axons (Study 3, Fig. 1A). Studies were conducted with 4-AP (0.5 mg/kg; i.p.) or saline vehicle injections initiated at 24 h after TBI or sham procedures and continued twice daily through day 6. Brains were collected for electrophysiology on day 7 after an overnight period for washout of circulating 4-AP in order to focus evaluation on the potential functional effects of the observed structural and molecular changes. The compound action potentials (CAPs) recorded using graded current intensities reflect the summed response of a population of axons successfully conducting from the stimulus electrode across the CC midline to reach the recording electrode (Fig. 6A). In the CC, CAPs form two distinct wave peaks based on the conduction velocity (Fig. 6B, C). As shown in the representative traces (Fig. 6C), the first wave to reach the recording electrode (N1) represents the response of fast-conducting myelinated axons, while the second wave (N2) comprises the response of slower conducting unmyelinated axons and may include demyelinated axons. TBI slowed conduction velocities in both the N1 and N2 axon populations (Fig. 6B). Acute 4-AP treatment did not alter conduction velocity in sham or TBI mice, as compared to the vehicle condition. The amplitude of the N1 CAP field potential appeared noticeably diminished in recordings from TBI animals compared to sham controls (Fig. 6C). Plotting the CAP amplitude against stimulus intensity revealed that the N1 CAP amplitude was consistently lower in mice that received a TBI as compared to the sham controls (Fig. 6D). The N2 CAP amplitude was not altered by TBI or 4-AP treatment (Fig. 6D). However, TBI did affect the shape of the N2 wave resulting in a widened waveform that returned to baseline more slowly (Fig. 6E-G). This broader range of axon velocities in the N2 wave, particularly those with slower conduction velocity, may reflect slowed conduction of unmyelinated axons. In addition, axons from the N1 wave that have impaired conduction due to demyelination may also slow so much as to fall back into the N2 wave. Demyelinated N1 axons within the N2 wave may mask the ability of the N2 CAP amplitude (Fig. 6D) to reveal loss of unmyelinated axons, which are expected to be vulnerable to TBI [61]. The shape of the N1 wave was not altered by TBI or 4-AP (data not shown).

**Fig. 7:**
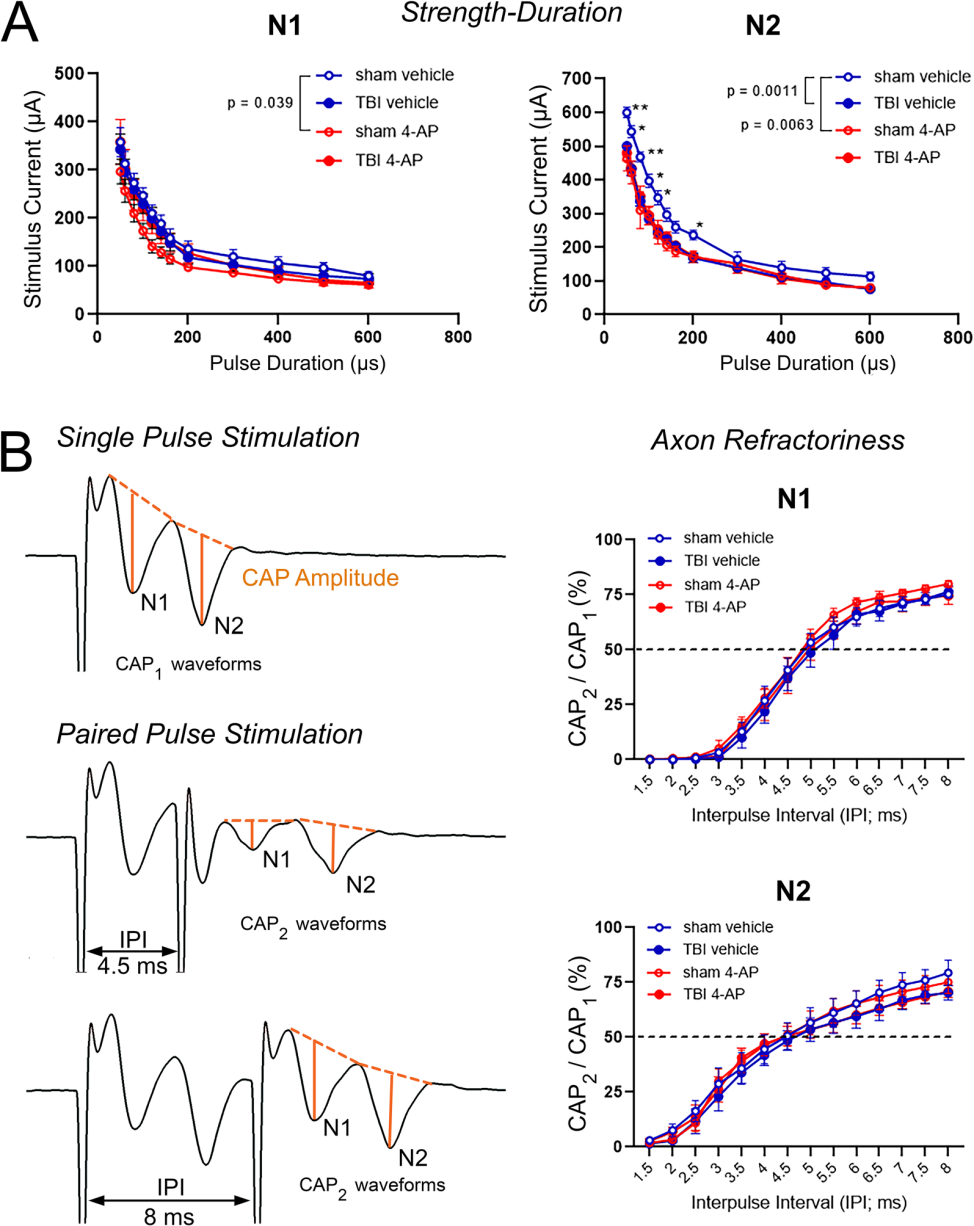
TBI alters the axon population excitability without changing the refractory period. A subset of mice had extended studies of additional conduction properties from the ex vivo brain slice preparations as shown in Fig. 6. **A** The axon intrinsic excitability was probed by measuring the stimulus duration relative to increasing stimulus current. In both N1 and N2 axons, 4-AP treatment shifted the strength-duration curve toward a more excitable state in sham mice, based on eliciting a similar response with a lower stimulus strength at a given stimulus duration. This strength-duration analysis also revealed that TBI induced hyperexcitability of N2 axons in the vehicle condition. The N2 axon hyperexcitability after TBI was similar in the 4-AP and vehicle conditions. **B** The axon refractory period due to recovery time between action potentials was probed with a paired-pulse protocol. Representative N1 and N2 compound action potential (CAP) waveforms from a single pulse stimulation as compared to a paired-pulse protocol with varying interpulse intervals (IPI). Orange lines from CAP peaks to their projected bases indicate the amplitude of each waveform. Plots of the percent ratio of each second pulse CAP amplitude (CAP_2_), evoked in the paired-pulse stimulation, divided by the CAP amplitude of the single reference pulse stimulation (CAP_1_) showed no differences in refractoriness between sham and TBI animals or between vehicle and 4-AP treatments. **A–B** Linear effects model statistical analysis and Holm-Sidak’s multiple comparison test (*p < 0.05, **p < 0.01). Data is expressed as mean ± SEM with n = 6–7 animals per group

Further analysis tested whether TBI and/or systemic 4-AP treatment altered the intrinsic excitability or refractory period of CC axons (Fig. 7). Following CAP amplitude and velocity recordings, additional recordings examined evoked CAP strength-duration properties. The threshold for activation of nerve fibers depends not only on stimulus strength, as shown with CAP amplitude analysis, but also on the electric charge from the product of the strength and duration of the stimulus. In order to probe the relationship between minimal intensity (current) of an electrical pulse and stimulus pulse duration, strength-duration testing was conducted as previously described [61]. This analysis showed that TBI significantly altered the strength-duration characteristics of the N2 CAP waveform at shorter pulse durations without affecting the excitability of the N1 wave (Fig. 7A). The leftward shift in the plot of stimulus current versus stimulus duration for sham vehicle compared to TBI vehicle animals suggests that slower conducting (N2) axons are more excitable following TBI. We also found that 4-AP treatment increased the excitability of both the N1 and N2 components of the CAP in sham mice, but not in TBI animals (Fig. 7A). This result suggests that in vivo 4-AP exposure decreases the stimulation threshold of CC axons, so less electric charge is sufficient to bring responsive fibers to threshold. Importantly, 4-AP did not exacerbate axonal excitability properties following TBI.

Finally, the refractory properties of CC axons were analyzed using a paired pulse paradigm in the brain slices as for strength-duration testing. After firing an action potential, axons enter a refractory period of reduced excitability. Axon refractoriness reflects the minimal interpulse interval needed to depolarize the membrane from a hyperpolarized state into a threshold capable of initiating a second action potential. Juxtaparanode Kv1 channels contribute to repolarize axons between impulses, which may be disrupted during demyelination [12, 18, 60]. To analyze axon refractoriness, the amplitudes of electrically evoked CAPs elicited by a paired pulse stimulation (CAP_2_ waveforms) were compared to the amplitude of their respective N1 and N2 CAP components from a single pulse stimulation (CAP_1_ waveforms) (Fig. 7B). Neither the TBI nor 4-AP treatment affected the refractoriness of the N1 or the N2 wave components. We can only speculate as to how this lack of change in the refractory period may correspond with the observed axon pathology. After TBI in human post-mortem cases, and in the current model, traumatic axonal injury appears in a pattern of damaged axons dispersed among intact adjacent axons in white matter tracts [54, 67, 72]. Possibly, the refractory period analysis may be indicative of the intact axon response, but may not be sensitive to the poorly conducting axons since CAP_2_ is normalized to CAP_1_.

Taken together, our electrophysiological results indicate that the main functional deficits after TBI impact the speed and population size of myelinated axons that conduct impulses across the CC, which was not altered by acute 4-AP treatment when examined on 7 day post-TBI.

## Discussion

The current pre-clinical study is the first test of 4-AP used as an acute treatment for TBI, and our results show significant benefit for reducing axon damage. 4-AP treatment initiated at a clinically reasonable time of one day after a TBI was effective at a low clinically relevant dosage that produced robust beneficial effects on CC axon and myelin pathology at one week after TBI. 4-AP significantly reduced axon damage using two distinct techniques in separate studies (Figs. 1, 2, 3,4). Using axonal YFP accumulations as a sensitive screen of axon damage showed the most robust 4-AP effect sizes, and demonstrated similar results in both sexes (Fig. 2). Significant positive 4-AP effects on axonal pathology were also identified based on mitochondrial swelling, impaired vesicle transport, and cytoskeletal compaction (Fig. 4). Furthermore, 4-AP significantly reduced axonal demyelination and disruption of nodal regions after TBI (Figs. 3, 4). These benefits of acute 4-AP treatment were found in a closed head TBI model that produced dispersed damaged axons distributed among intact axons within the white matter [54, 72], reflecting the hallmark pathological characteristics of diffuse axonal injury in cases of human TBI [67, 68].

Our results also suggest that 4-AP treatment enhances repair of multiple myelination-related outcomes. At 7 days post-TBI, myelin sheaths were abnormally thin as compared to sham values in both 4-AP and vehicle cohorts (Fig. 5), a feature associated with remyelination and preservation of axonal function in experimental demyelination [25]. Consistent with ongoing remyelination, demyelination decreased significantly between 3 and 7 days after TBI (p = 0.0066 vehicle; *p* = 0.0004 4-AP), but it was only in the 4-AP treated group that demyelination returned to sham levels (Fig. 4). Furthermore, heminodes have been shown to form during developmental myelination or where remyelination is incomplete [8, 14, 33], and the increased frequency of heminodes after TBI was reduced to sham levels only in the 4-AP treated mice (Fig. 3). Interpreting the 4-AP reduction of heminodes as reparative is supported by our prior study that showed increased heminode frequency occurs by at least 3 days post-TBI and did not resolve but was instead further increased at 6 weeks Marion et al. [50]. Collectively, these findings (Figs. 2–5) suggest that TBI causes axon damage with myelin sheath detachment from paranodes and demyelinated axon segments, and that 4-AP treatment enhances recovery from this damage.

The outcomes of our studies provide further evidence that treatment of acute neurological injuries with 4-AP provides multiple unexpected benefits. In acute peripheral nerve crush injuries in mice, initiation of treatment with clinically relevant concentrations of 4-AP one day post-injury promoted remyelination and enhanced axonal area Tseng et al. [74]. 4-AP treatment has also been reported to prevent onset of experimental allergic enchephalomyelitis and decrease damage from optic nerve crush in mice Dietrich et al. [21], but these studies initiated 4-AP one week prior to immunization or injury, with daily dosages more than tenfold greater than those shown to provide clinically relevant serum concentrations of 4-AP in the current study (Fig. 1) and in oral treatment of peripheral nerve injury Hsu et al. [39].

The mechanisms by which relatively short-term treatment with 4-AP, initiated 24 h post-injury at clinically relevant dosages, enhances recovery in acute neurological injuries are unknown, and may be multi-faceted. 4-AP is thought to inhibit Kv channels but the overall result also depends on sodium and calcium channel activity, and is hard to predict at low 4-AP concentrations [1, 20, 81]. Beyond potential 4-AP actions at nodal regions, low 4-AP levels may have an activity dependent effect on axon and myelin health from Kv channel inhibition that potentiates synaptic transmission to enhance axon and neural circuit activity [66, 69]. 4-AP has also shown symptomatic benefit to improve the function of regenerating axons [4, 47]. Finally, 4-AP mechanisms in TBI may involve potassium channels on multiple cell types, including neurons, glia, or immune cells, and pathological conditions change the pattern of potassium channel expression and localization [5, 10, 20, 40, 42, 62].

Despite the multiple benefits of 4-AP treatment on axon pathology in our TBI studies, the results differ from outcomes on peripheral nerve injury with respect to recovery of nerve function. However, the electrophysiological deficits of CC axons observed after TBI (Fig. 6) are consistent with the structural aspects that were not altered by 4-AP treatment. The N1 axon conduction velocity is dependent on axon diameter and myelin thickness [77], which were reduced after TBI (Fig. 5). The lower N1 CAP amplitude indicates that fewer axons conducted signal through the CC after TBI due to loss of damaged axons or impaired conduction along viable axons [24, 32, 75]. These electrophysiological findings are in agreement with prior studies in CC axons after TBI from our lab and others [22, 50, 61–63]. The lack of 4-AP benefit on axon conduction properties could have been because TBI is a more complex injury than peripheral nerve crush or/and because more time is needed to observe benefits on these parameters.

The current results focus on the critical first week after TBI, when axons are highly vulnerable, and definitively show that 4-AP is acting during the acute phase to reduce axon pathology. Evaluating functional improvement with 4-AP treatment after TBI may require longer term studies and/or additional approaches. Our electrophysiological analyses approach provided a direct measure of conduction properties among the CC axon population, but can only detect changes involving a large number of axons that achieve a capability threshold of successfully conducting action potentials across the CC. Action potential transmission may fail in surviving or recovering axons due to impedance mismatch between myelinated and incompletely remyelinated areas, or, due to current shunting at unstable axon-myelin paranode junctions [12, 76], which may be factors at our 7 day time point. During remyelination, proper Kv1 localization in juxtaparanodes occurs at a late stage of node formation [60, 89] and Kv1.2 localization to juxtaparanodes was significantly improved with 4-AP treatment but had not recovered to sham levels (Fig. 3). Interestingly, previous animal and human studies indicate functional improvement may require longer duration studies of 4-AP treatment. Low dose 4-AP treatment initiated one day after peripheral nerve crush resulted in significant gait improvement at 3 days post-injury that progressed through 8 days, although a significant increase in nerve conduction velocity was not observed until 21 days [74]. Furthermore, clinical trials of 4-AP (dalfampridine) for patients with chronic MS used a treatment duration of 4 weeks or more to evaluate improvement of function [87].

Fully understanding the mechanism(s) by which 4-AP treatment decreases damage and enhances recovery after TBI or peripheral nerve injury will require further challenging studies. It is perhaps more important that repurposing of 4-AP for examination in these traumatic injuries is more straightforward. Due to the long-standing interest in the use of 4-AP in treating multiple chronic neurological problems (including MS, nystagmus, Lambert-Easton myasthenic syndrome and spinal cord injury), extensive information exists regarding appropriate dosage regimens, side-effects and other concerns related to translation of studies from the laboratory to the clinic [17]. Studies on diagnosis and recovery from peripheral nerve injury have already led to two clinical trials on 4-AP treatment of acute injuries [28, 29]. A recent report of 4-AP treatment in patients with KCNA2-encephalopathy has interesting mechanistic implications; 4-AP reduced seizures in patients with gain-of-function variants of the Kv1.2 subunit and antagonized the electrophysiological defects in vitro in transfected neurons that expressed variant Kv1.2 channels [37]. In addition, methods to evaluate 4-AP target engagement and potential mechanism(s) of action in the brain may be facilitated by development of a 4-AP radioligand for neuroimaging, which has detected cortical pathology from brain injury in nonhuman primate testing and has advanced to a clinical trial [9, 36]. The need for treatments for acute TBI and the promising results of our present study suggest the importance of repurposing 4-AP for TBI as a new clinical indication.

Several limitations should be considered in interpreting these findings. Low dose 4-AP was administered twice daily, as for dalfampridine, without the extended release achieved in the capsule formulation. Higher 4-AP dosing may not be clinically reasonable due to adverse effects, but extended release methods to prolong the 4-AP exposure would be of interest for further testing in TBI. This initial study focused on white matter injury, specifically CC axon and myelin pathology, whereas 4-AP effects may also involve glial cells or peripheral immune cells [20]. Broader 4-AP effects may involve neural circuit and synaptic properties, and the fidelity of high frequency signal transmission [45, 83, 86, 87], which may require more extensive electrophysiological studies.

## Conclusion

This first test of the effects of 4-AP treatment in TBI demonstrates significantly reduced axon pathology using multiple measures. This study focused on use of 4-AP as an acute treatment, which was initiated at a clinically reasonable time of one day after the experimental TBI. Preserving and/or enhancing recovery of surviving axons at the acute stage should have the greatest potential to prevent persistent symptoms. 4-AP treatment after TBI may also have the potential to attenuate neurodegeneration based on retrospective evidence in patients with MS [21]. An effective treatment to reduce axon damage is an important target to improve outcomes for patients who experience a TBI.

## Data Availability

Pre-registered study plans can be found on Open Science Framework (https://osf.io/registries). The datasets used and/or analyzed during the current study are available from the corresponding author on reasonable request.

## Abbreviations

4-AP: 4-Aminopyridine
ACSF: Artificial cerebral spinal fluid
ANOVA: Analysis of variance
AP: Anterior–posterior
CAP: Compound action potential
CC: Corpus callosum
Cing: Cingulum
DV: Dorso-ventral
ED_50_: Median effective dose
EM: Electron microscopy
IPI: Interpulse interval
Kv: Voltage-gated potassium channel
Kv1.2: Voltage-gated potassium channel subfamily A member 2
LC**–**MS/MS: Liquid chromatography with tandem mass spectrometry
LSM: Laser scanning microscope
LV: Lateral ventricle
MIP: Maximum intensity projection
ML: Medial-lateral
MS: Multiple sclerosis
N1: Compound action potential from myelinated axons
N2: Compound action potential from de/unmyelinated axons
PFA: Paraformaldehyde
RCT: Randomized controlled trial
ROI: Region of interest
SEM: Standard error of mean
TBI: Traumatic brain injury
YFP: Yellow fluorescent protein

## References

[1] Agren R, Nilsson J, Arhem P (2019). Closed and open state dependent block of potassium channels cause opposing effects on excitability - a computational approach. Sci Rep.

[2] Andravizou A, Dardiotis E, Artemiadis A, Sokratous M, Siokas V, Tsouris Z, Aloizou AM, Nikolaidis I, Bakirtzis C, Tsivgoulis G (2019). Brain atrophy in multiple sclerosis: mechanisms, clinical relevance and treatment options. Auto Immun Highlights.

[3] Balan IS, Saladino AJ, Aarabi B, Castellani RJ, Wade C, Stein DM, Eisenberg HM, Chen HH, Fiskum G (2013). Cellular alterations in human traumatic brain injury: changes in mitochondrial morphology reflect regional levels of injury severity. J Neurotrauma.

[4] Bei F, Lee HHC, Liu X, Gunner G, Jin H, Ma L, Wang C, Hou L, Hensch TK, Frank E (2016). Restoration of visual function by enhancing conduction in regenerated axons. Cell.

[5] Boscia F, Elkjaer ML, Illes Z, Kukley M (2021). Altered expression of ion channels in white matter lesions of progressive multiple sclerosis: what do we know about their function?. Front Cell Neurosci.

[6] Bradshaw DV, Kim Y, Fu A, Marion CM, Radomski KL, McCabe JT, Armstrong RC (2021). Repetitive blast exposure produces white matter axon damage without subsequent myelin remodeling: in vivo analysis of brain injury using fluorescent reporter mice. Neurotrauma Rep.

[7] Bradshaw DV, Knutsen AK, Korotcov A, Sullivan GM, Radomski KL, Dardzinski BJ, Zi X, McDaniel DP, Armstrong RC (2021). Genetic inactivation of SARM1 axon degeneration pathway improves outcome trajectory after experimental traumatic brain injury based on pathological, radiological, and functional measures. Acta Neuropathol Commun.

[8] Brivio V, Faivre-Sarrailh C, Peles E, Sherman DL, Brophy PJ (2017). Assembly of CNS nodes of ranvier in myelinated nerves is promoted by the axon cytoskeleton. Curr Biol.

[9] Brugarolas P (2021) Investigating the Utility of Demyelination Tracer 18F 3F4AP in Controls and Multiple Sclerosis Subjects

[10] Calvo M, Richards N, Schmid AB, Barroso A, Zhu L, Ivulic D, Zhu N, Anwandter P, Bhat MA, Court FA (2016). Altered potassium channel distribution and composition in myelinated axons suppresses hyperexcitability following injury. eLife.

[11] Capacio BR, Byers CE, Matthews RL, Chang FC (1996). A method for determining 4-aminopyridine in plasma: pharmacokinetics in anaesthetized guinea pigs after intravenous administration. Biomed Chromatogr.

[12] Cohen CCH, Popovic MA, Klooster J, Weil MT, Mobius W, Nave KA, Kole MHP (2020). Saltatory conduction along myelinated axons involves a periaxonal nanocircuit. Cell.

[13] Cohen J (1988) Statistical power analysis for the behavioral sciences. Lawrence Erlbaum Associates, City

[14] Coman I, Aigrot MS, Seilhean D, Reynolds R, Girault JA, Zalc B, Lubetzki C (2006). Nodal, paranodal and juxtaparanodal axonal proteins during demyelination and remyelination in multiple sclerosis. Brain.

[15] Crawford DK, Mangiardi M, Tiwari-Woodruff SK (2009). Assaying the functional effects of demyelination and remyelination: revisiting field potential recordings. J Neurosci Methods.

[16] Dams-O'Connor K, Spielman L, Singh A, Gordon WA, Lingsma HF, Maas AI, Manley GT, Mukherjee P, Okonkwo DO, Puccio AM (2013). The impact of previous traumatic brain injury on health and functioning: a TRACK-TBI study. J Neurotrauma.

[17] De Giglio L, Cortese F, Pennisi EM (2020). Aminopiridines in the treatment of multiple sclerosis and other neurological disorders. Neurodegener Dis Manag.

[18] Devaux J, Gola M, Jacquet G, Crest M (2002). Effects of K+ channel blockers on developing rat myelinated CNS axons: identification of four types of K+ channels. J Neurophysiol.

[19] Dewitt DS, Perez-Polo R, Hulsebosch CE, Dash PK, Robertson CS (2013). Challenges in the development of rodent models of mild traumatic brain injury. J Neurotrauma.

[20] Dietrich M, Hartung HP, Albrecht P (2021). Neuroprotective properties of 4-aminopyridine. Neurol Neuroimmunol Neuroinflamm.

[21] Dietrich M, Koska V, Hecker C, Gottle P, Hilla AM, Heskamp A, Lepka K, Issberner A, Hallenberger A, Baksmeier C (2020). Protective effects of 4-aminopyridine in experimental optic neuritis and multiple sclerosis. Brain.

[22] Dileonardi AM, Huh JW, Raghupathi R (2012). Differential effects of FK506 on structural and functional axonal deficits after diffuse brain injury in the immature rat. J Neuropathol Exp Neurol.

[23] Donders J, Strong CA (2014). Clinical utility of the wechsler adult intelligence scale-fourth edition after traumatic brain injury. Assessment.

[24] Duncan GJ, Simkins TJ, Emery B (2021). Neuron-oligodendrocyte interactions in the structure and integrity of axons. Front Cell Dev Biol.

[25] Duncan ID, Marik RL, Broman AT, Heidari M (2017). Thin myelin sheaths as the hallmark of remyelination persist over time and preserve axon function. Proc Natl Acad Sci U S A.

[26] Dutta S, Sengupta P (2016). Men and mice: Relating their ages. Life Sci.

[27] Dymowski AR, Ponsford JL, Willmott C (2016). Cognitive training approaches to remediate attention and executive dysfunction after traumatic brain injury: A single-case series. Neuropsychol Rehabil.

[28] Elfar J (2021) 4-aminopyridine Treatment for Nerve Injury. Identifier NCT03701581. https://ClinicalTrials.gov/show/NCT03701581

[29] Elfar J (2021) A single dose pharmaco-diagnostic for peripheral nerve continuity after trauma. Identifier NCT04026568. https://ClinicalTrials.gov/show/NCT04026568

[30] Feng G, Mellor RH, Bernstein M, Keller-Peck C, Nguyen QT, Wallace M, Nerbonne JM, Lichtman JW, Sanes JR (2000). Imaging neuronal subsets in transgenic mice expressing multiple spectral variants of GFP. Neuron.

[31] Filley CM, Kelly JP (2018). White matter and cognition in traumatic brain injury. J Alzheimers Dis.

[32] Franssen H (2008). Electrophysiology in demyelinating polyneuropathies. Expert Rev Neurother.

[33] Freeman SA, Desmazieres A, Fricker D, Lubetzki C, Sol-Foulon N (2016). Mechanisms of sodium channel clustering and its influence on axonal impulse conduction. Cell Mol Life Sci.

[34] Goodman AD, Stone RT (2013). Enhancing neural transmission in multiple sclerosis (4-aminopyridine therapy). Neurotherapeutics.

[35] Gu Y, Jukkola P, Wang Q, Esparza T, Zhao Y, Brody D, Gu C (2017). Polarity of varicosity initiation in central neuron mechanosensation. J Cell Biol.

[36] Guehl NJ, Neelamegam R, Zhou YP, Moon SH, Dhaynaut M, El Fakhri G, Normandin MD, Brugarolas P (2021). Radiochemical synthesis and evaluation in Non-human primates of 3-[(11)C]methoxy-4-aminopyridine: a novel PET tracer for imaging potassium channels in the CNS. ACS Chem Neurosci.

[37] Hedrich UBS, Lauxmann S, Wolff M, Synofzik M, Bast T, Binelli A, Serratosa JM, Martinez-Ulloa P, Allen NM, King MD (2021). 4-Aminopyridine is a promising treatment option for patients with gain-of-function KCNA2-encephalopathy. Sci Transl Med.

[38] Henderson VC, Kimmelman J, Fergusson D, Grimshaw JM, Hackam DG (2013). Threats to validity in the design and conduct of preclinical efficacy studies: a systematic review of guidelines for in vivo animal experiments. PLoS Med.

[39] Hsu CG, Talukder MAH, Yue L, Turpin LC, Noble M, Elfar JC (2020). Human equivalent dose of oral 4-aminopyridine differentiates nerve crush injury from transection injury and improves post-injury function in mice. Neural Regen Res.

[40] Judge SI, Bever CT (2006). Potassium channel blockers in multiple sclerosis: neuronal Kv channels and effects of symptomatic treatment. Pharmacol Ther.

[41] Juengst SB, Nabasny A, Terhorst L (2020). Cohort differences in neurobehavioral symptoms in chronic mild to severe traumatic brain injury. Front Neurol.

[42] Jukkola P, Gu Y, Lovett-Racke AE, Gu C (2017). Suppression of inflammatory demyelinaton and axon degeneration through inhibiting Kv3 channels. Front Mol Neurosci.

[43] Kilkenny C, Browne WJ, Cuthill IC, Emerson M, Altman DG (2010). Improving bioscience research reporting: the ARRIVE guidelines for reporting animal research. PLoS Biol.

[44] Koliatsos VE, Alexandris AS (2019). Wallerian degeneration as a therapeutic target in traumatic brain injury. Curr Opin Neurol.

[45] Lang-Ouellette D, Gruver KM, Smith-Dijak A, Blot FGC, Stewart CA, de Vanssay-de-Blavous P, Li CH, Van Eitrem C, Rosen C, Faust PL (2021). Purkinje cell axonal swellings enhance action potential fidelity and cerebellar function. Nat Commun.

[46] Levin HS, Temkin NR, Barber J, Nelson LD, Robertson C, Brennan J, Stein MB, Yue JK, Giacino JT, McCrea MA (2021). Association of sex and age with mild traumatic brain injury-related symptoms: a TRACK-TBI study. JAMA Netw Open.

[47] Liu Y, Wang X, Li W, Zhang Q, Li Y, Zhang Z, Zhu J, Chen B, Williams PR, Zhang Y (2017). A sensitized IGF1 treatment restores corticospinal axon-dependent functions. Neuron.

[48] Loring HS, Thompson PR (2020). Emergence of SARM1 as a potential therapeutic target for wallerian-type diseases. Cell Chem Biol.

[49] Marion CM, McDaniel DP, Armstrong RC (2019). Sarm1 deletion reduces axon damage, demyelination, and white matter atrophy after experimental traumatic brain injury. Exp Neurol.

[50] Marion CM, Radomski KL, Cramer NP, Galdzicki Z, Armstrong RC (2018). Experimental traumatic brain injury identifies distinct early and late phase axonal conduction deficits of white matter pathophysiology, and reveals intervening recovery. J Neurosci.

[51] Marmarou CR, Walker SA, Davis CL, Povlishock JT (2005). Quantitative analysis of the relationship between intra- axonal neurofilament compaction and impaired axonal transport following diffuse traumatic brain injury. J Neurotrauma.

[52] Maxwell W, Bartlet E, Morgan H (2014). Wallerian degeneration in the optic nerve stretch-injury model of TBI: a stereological analysis. J Neurotrauma.

[53] McCrea MA, Giacino JT, Barber J, Temkin NR, Nelson LD, Levin HS, Dikmen S, Stein M, Bodien YG, Boase K (2021). Functional outcomes over the first year after moderate to severe traumatic brain injury in the prospective, longitudinal TRACK-TBI study. JAMA Neurol.

[54] Mierzwa AJ, Marion CM, Sullivan GM, McDaniel DP, Armstrong RC (2015). Components of myelin damage and repair in the progression of white matter pathology after mild traumatic brain injury. J Neuropathol Exp Neurol.

[55] Mierzwa AJ, Sullivan GM, Beer LA, Ahn S, Armstrong RC (2014). Comparison of cortical and white matter traumatic brain injury models reveals differential effects in the subventricular zone and divergent sonic hedgehog signaling pathways in neuroblasts and oligodendrocyte progenitors. ASN Neuro.

[56] Morehead M, Bartus RT, Dean RL, Miotke JA, Murphy S, Sall J, Goldman H (1994). Histopathologic consequences of moderate concussion in an animal model: correlations with duration of unconsciousness. J Neurotrauma.

[57] Nikic I, Merkler D, Sorbara C, Brinkoetter M, Kreutzfeldt M, Bareyre FM, Bruck W, Bishop D, Misgeld T, Kerschensteiner M (2011). A reversible form of axon damage in experimental autoimmune encephalomyelitis and multiple sclerosis. Nat Med.

[58] Pan S, Chan JR (2017). Regulation and dysregulation of axon infrastructure by myelinating glia. J Cell Biol.

[59] Pernici CD, Rowe RK, Doughty PT, Madadi M, Lifshitz J, Murray TA (2020). Longitudinal optical imaging technique to visualize progressive axonal damage after brain injury in mice reveals responses to different minocycline treatments. Sci Rep.

[60] Rasband MN, Trimmer JS, Schwarz TL, Levinson SR, Ellisman MH, Schachner M, Shrager P (1998). Potassium channel distribution, clustering, and function in remyelinating rat axons. J Neurosci.

[61] Reeves TM, Phillips LL, Povlishock JT (2005). Myelinated and unmyelinated axons of the corpus callosum differ in vulnerability and functional recovery following traumatic brain injury. Exp Neurol.

[62] Reeves TM, Smith TL, Williamson JC, Phillips LL (2012). Unmyelinated axons show selective rostrocaudal pathology in the corpus callosum after traumatic brain injury. J Neuropathol Exp Neurol.

[63] Reeves TM, Trimmer PA, Colley BS, Phillips LL (2016). Targeting Kv1.3 channels to reduce white matter pathology after traumatic brain injury. Exp Neurol.

[64] Roseborough A, Hachinski V, Whitehead S (2020). White matter degeneration-A treatable target?. JAMA Neurol.

[65] Simon DJ, Watkins TA (2018). Therapeutic opportunities and pitfalls in the treatment of axon degeneration. Curr Opin Neurol.

[66] Sindhurakar A, Mishra AM, Gupta D, Iaci JF, Parry TJ, Carmel JB (2017). Clinically relevant levels of 4-aminopyridine strengthen physiological responses in intact motor circuits in rats, especially after pyramidal tract injury. Neurorehabil Neural Repair.

[67] Smith DH, Hicks R, Povlishock JT (2013). Therapy development for diffuse axonal injury. J Neurotrauma.

[68] Smith DH, Stewart W (2020). 'Concussion' is not a true diagnosis. Nat Rev Neurol.

[69] Smith KJ, Felts PA, John GR (2000). Effects of 4-aminopyridine on demyelinated axons, synapses and muscle tension. Brain.

[70] Sturrock RR (1976). Changes in neurologia and myelination in the white matter of aging mice. J Gerontol.

[71] Sturrock RR (1980). Myelination of the mouse corpus callosum. Neuropathol Appl Neurobiol.

[72] Sullivan GM, Mierzwa AJ, Kijpaisalratana N, Tang H, Wang Y, Song SK, Selwyn R, Armstrong RC (2013). Oligodendrocyte lineage and subventricular zone response to traumatic axonal injury in the corpus callosum. J Neuropathol Exp Neurol.

[73] Taylor CA, Bell JM, Breiding MJ, Xu L (2017). Traumatic brain injury-related emergency department visits, hospitalizations, and deaths - United States, 2007 and 2013. MMWR Surveill Summ.

[74] Tseng KC, Li H, Clark A, Sundem L, Zuscik M, Noble M, Elfar J (2016). 4-Aminopyridine promotes functional recovery and remyelination in acute peripheral nerve injury. EMBO Mol Med.

[75] Uncini A, Santoro L (2020). The electrophysiology of axonal neuropathies: More than just evidence of axonal loss. Clin Neurophysiol.

[76] Utzschneider DA, Archer DR, Kocsis JD, Waxman SG, Duncan ID (1994). Transplantation of glial cells enhances action potential conduction of amyelinated spinal cord axons in the myelin-deficient rat. Proc Natl Acad Sci U S A.

[77] Waxman SG (1980). Determinants of conduction velocity in myelinated nerve fibers. Muscle Nerve.

[78] Weber MT, Arena JD, Xiao R, Wolf JA, Johnson VE (2019). CLARITY reveals a more protracted temporal course of axon swelling and disconnection than previously described following traumatic brain injury. Brain Pathol.

[79] Williams PR, Marincu BN, Sorbara CD, Mahler CF, Schumacher AM, Griesbeck O, Kerschensteiner M, Misgeld T (2014). A recoverable state of axon injury persists for hours after spinal cord contusion in vivo. Nat Commun.

[80] Witte ME, Schumacher AM, Mahler CF, Bewersdorf JP, Lehmitz J, Scheiter A, Sanchez P, Williams PR, Griesbeck O, Naumann R (2019). Calcium influx through plasma-membrane nanoruptures drives axon degeneration in a model of multiple sclerosis. Neuron.

[81] Wu ZZ, Li DP, Chen SR, Pan HL (2009). Aminopyridines potentiate synaptic and neuromuscular transmission by targeting the voltage-activated calcium channel beta subunit. J Biol Chem.

[82] Yamaguchi S, Rogawski MA (1992). Effects of anticonvulsant drugs on 4-aminopyridine-induced seizures in mice. Epilepsy Res.

[83] Yang YM, Wang W, Fedchyshyn MJ, Zhou Z, Ding J, Wang LY (2014). Enhancing the fidelity of neurotransmission by activity-dependent facilitation of presynaptic potassium currents. Nat Commun.

[84] Yu F, Shukla DK, Armstrong RC, Marion CM, Radomski KL, Selwyn RG, Dardzinski BJ (2017). Repetitive model of mild traumatic brain injury produces cortical abnormalities detectable by magnetic resonance diffusion imaging (DTI/DKI), histopathology, and behavior. J Neurotrauma.

[85] Yue JK, Phelps RR, Hemmerle DD, Upadhyayula PS, Winkler EA, Deng H, Chang D, Vassar MJ, Taylor SR, Schnyer DM (2021). Predictors of six-month inability to return to work in previously employed subjects after mild traumatic brain injury: a TRACK-TBI pilot study. J Concussion.

[86] Zang Y, Marder E (2021). Interactions among diameter, myelination, and the Na/K pump affect axonal resilience to high-frequency spiking. Proc Natl Acad Sci U S A.

[87] Zhang E, Tian X, Li R, Chen C, Li M, Ma L, Wei R, Zhou Y, Cui Y (2021). Dalfampridine in the treatment of multiple sclerosis: a meta-analysis of randomised controlled trials. Orphanet J Rare Dis.

[88] Zhou YX, Pannu R, Le TQ, Armstrong RC (2012). Fibroblast growth factor 1 (FGFR1) modulation regulates repair capacity of oligodendrocyte progenitor cells following chronic demyelination. Neurobiol Dis.

[89] Zoupi L, Markoullis K, Kleopa KA, Karagogeos D (2013). Alterations of juxtaparanodal domains in two rodent models of CNS demyelination. Glia.

